# Frequent silencing of the candidate tumor suppressor *TRIM58* by promoter methylation in early-stage lung adenocarcinoma

**DOI:** 10.18632/oncotarget.13761

**Published:** 2016-12-01

**Authors:** Koichiro Kajiura, Kiyoshi Masuda, Takuya Naruto, Tomohiro Kohmoto, Miki Watabnabe, Mitsuhiro Tsuboi, Hiromitsu Takizawa, Kazuya Kondo, Akira Tangoku, Issei Imoto

**Affiliations:** ^1^ Department of Human Genetics, Graduate School of Biomedical Sciences, Tokushima University, Tokushima, Japan; ^2^ Department of Thoracic, Endocrine and Oncological Surgery, Graduate School of Biomedical Sciences, Tokushima University, Tokushima, Japan; ^3^ Department of Oncological Medical Services, Graduate School of Biomedical Sciences, Tokushima University, Tokushima, Japan

**Keywords:** TRIM58, early-stage lung adenocarcinoma, tumor suppressor gene, methylation, smoking status

## Abstract

In this study, we aimed to identify novel drivers that would be epigenetically altered through aberrant methylation in early-stage lung adenocarcinoma (LADC), regardless of the presence or absence of tobacco smoking-induced epigenetic field defects. Through genome-wide screening for aberrantly methylated CpG islands (CGIs) in 12 clinically uniform, stage-I LADC cases affecting six non-smokers and six smokers, we identified candidate tumor-suppressor genes (TSGs) inactivated by hypermethylation. Through systematic expression analyses of those candidates in panels of additional tumor samples and cell lines treated or not treated with 5-aza-deoxycitidine followed by validation analyses of cancer-specific silencing by CGI hypermethylation using a public database, we identified *TRIM58* as the most prominent candidate for TSG. *TRIM58* was robustly silenced by hypermethylation even in early-stage primary LADC, and the restoration of TRIM58 expression in LADC cell lines inhibited cell growth *in vitro* and *in vivo* in anchorage-dependent and -independent manners. Our findings suggest that aberrant inactivation of *TRIM58* consequent to CGI hypermethylation might stimulate the early carcinogenesis of LADC regardless of smoking status; furthermore, *TRIM58* methylation might be a possible early diagnostic and epigenetic therapeutic target in LADC.

## INTRODUCTION

Lung adenocarcinoma (LADC) is the most predominant histologic subtype of lung cancer and one of the leading causes of cancer-related death worldwide [1, 2]. Although major improvements in treatment strategies have increased the short-term survival of patients with LADC, the impacts on long-term survival have remained modest [3]. Both late diagnosis and the scarcity of therapies that effectively achieve durable responses are important factors associated with poor outcomes. A better understanding of the molecular pathogenesis of LADC is needed to identify biomarkers that could be useful for early detection and to develop novel targeted therapies.

Aberrant promoter CpG island (CGI) methylation of tumor suppressor genes (TSGs) has been established as a common epigenetic mechanism underlying the pathogenesis of human cancers, including LADC [4, 5]. DNA methylation is a stable modification that often occurs at an early stage of carcinogenesis; as a result, such events are sensitive and specific biomarkers, even in tumor samples contaminated by non-tumorous cells as well as various biological fluids used for liquid biopsies. In LADC, distinct interactions between genetic and epigenetic changes were found to contribute differently to carcinogenesis in smokers (S) vs. never smokers (NS) [6, 7]. However, few genes that are altered by aberrant DNA methylation irrespective of etiology (particularly smoking status) and contribute to early LADC carcinogenesis have been identified.

In the present study, we performed a systematic, genome-wide screening of aberrantly methylated CGIs in stage-I LADCs from both S and NS to identify genes commonly dysregulated via aberrant DNA methylation in early-stage LADC, regardless of smoking status. To this end, we applied a series of criteria that targeted novel genes frequently silenced in LADC tumors and cell lines (possibly through hypermethylation) to prioritize the selection of top candidates for validation. *TRIM58*, a subsequently identified candidate, showed frequent CGI hypermethylation and its inverse relationship with gene expression in a larger set of LADC tumors and cell lines, followed by validation using a data set obtained from The Cancer Genome Atlas (TCGA). In addition, our functional analyses demonstrated the ubiquitin-ligase activity-dependent growth suppressive effect of TRIM58 on LADC cells both *in vitro* and *in vivo*, suggesting that this gene exerts TSG during the initiation and/or early progression stages of LADC.

## RESULTS

### Screening of aberrantly methylated and silenced genes from the early stage of LADC

**Figure 1:**
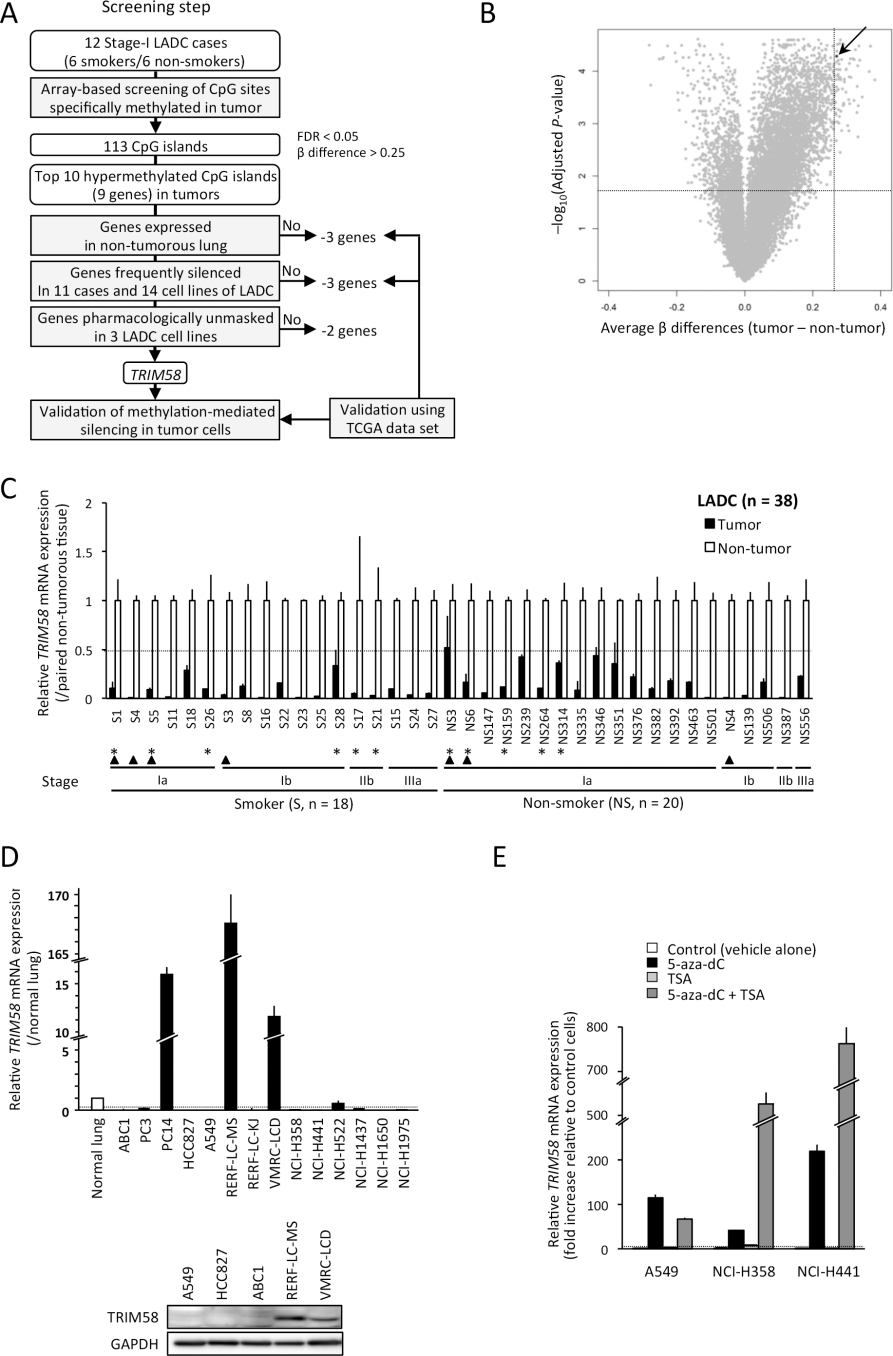
TRIM58 was identified as a candidate in a screening of genes silenced through hypermethylation of the CpG islands (CGI) in lung adenocarcinomas (LADCs) from an early stage, regardless of patient smoking status (**A**) Flow chart indicating the study design, which aimed to identify candidate tumor suppressor genes using experimental and bioinformatic filters. (**B**) Volcano plot of the differential CGI methylation statuses of 12 stage-I LADC tumors and paired non-tumorous tissues. The x-axis indicates the average difference in β-value (methylation level), and the y-axis indicates the–log10 of the adjusted *P*-value for each CGI. The arrow indicates the CGI around *TRIM58*. (**C**) Relative expression (fold change) of *TRIM58* mRNA in tumor tissues vs. paired non-tumorous tissues in a panel of LADC cases (*n* = 38 including 18 smokers [S] and 20 non-smokers [NS]; Supplementary Table S1) evaluated by qRT-PCR. Expression data were normalized to *GAPDH* (means ± standard deviations [SD] of triplicate experiments). Asterisks indicate 11 cases used to screen candidate gene expression (Supplementary Figure S2); arrowheads indicate cases used for array-based methylation screening (see Table 1). Dotted line indicates 0.5. (**D**) Expression levels of *TRIM58* mRNA (upper) and protein (lower) in LADC cell lines. Upper, relative expression levels of *TRIM58* mRNA in 14 LADC cell lines vs. normal lung tissues; expression was evaluated using qRT-PCR and normalized to *GAPDH*. Dotted line indicates 0.2. Lower, TRIM58 protein expression in five cell lines was determined by western blotting with a TRIM58-specific antibody. (**E**) Effect of treatment with 5-aza-dC and/or TSA on *TRIM58* mRNA expression in three LADC cell lines. The expression levels of *TRIM58* mRNA, which were evaluated by qRT-PCR and normalized to *GAPDH*, are shown relative to those of vehicle-treated control cells (means ± SDs of triplicate experiments). Dotted line indicates four (fold change).

We initially screened tumors and paired non-tumorous tissues obtained from 12 male patients with stage-I LADC (6 S, 6 NS; Table 1) using an Illumina HumanMethylation450K BeadChip to identify differentially methylated CGIs in a genome-wide manner (Figure 1A). Because the number of hypermethylated CGIs was much larger than that of hypomethylated CGIs (Figure 1B), we focused on the former group. A total of 113 CGIs were identified as differentially hypermethylated CGIs, with a false discovery rate (FDR) of < 0.05 and β difference (tumor − non-tumorous tissue) of > 0.25.

**Table 1:** Clinicopathological characteristics of patients with LADC analyzed by Infinium HumanMethylation450K BeadChips

ID	Age(yrs)	Gender	Smoking status	Pack-years	Tumor		
					TNM classification	Stage	Size (mm)
S1	68	Male	Smoker	1000	pT1aN0M0	Ia	13 × 11
S2	80	Male	Smoker	1200	pT1aN0M0	Ia	14 × 11
S3	60	Male	Smoker	1020	pT2aN0M0	Ib	17 × 11
S4	59	Male	Smoker	1600	pT1aN0M0	Ia	13 × 10
S5	74	Male	Smoker	2400	pT1aN0M0	Ia	12 × 11
S6	75	Male	Smoker	1000	pT1aN0M0	Ia	25 × 20
NS1	77	Male	Never smoker	0	pT1aN0M0	Ia	20 × 13
NS594	74	Male	Never smoker	0	pT1aN0M0	Ia	19 × 17
NS3	57	Male	Never smoker	0	pT1aN0M0	Ia	15 × 15
NS4	73	Male	Never smoker	0	pT2aN0M0	Ib	42 × 19
NS5	51	Male	Never smoker	0	pT1aN0M0	Ia	14 × 9
NS6	68	Male	Never smoker	0	pT1aN0M0	Ia	13 × 11

Next, we determined the mRNA expression of 9 genes associated with the top 10 CGIs (Table 2) in commercially available normal lung, panels of 11 stage-I or -II LADC cases (Supplementary Table S1), and 14 LADC cell lines via quantitative reverse-transcription polymerase chain reaction (qRT-PCR). Differential hypermethylation in tumors of those genes except *MEIS2* was validated using 29 paired cases of a TCGA data set (Supplementary Table S2). Three genes (*DDX25*, *FEZF2*, and *C12orf42*) that exhibited no or very low expression in commercially-available normal lung and non-tumorous lung tissues (data not shown) as well as 58 non-tumorous lung tissues from the TCGA data set (Supplementary Figure S1). Three genes (*ZNF577*, *MEIS2*, and *PTPRN2*) were infrequently downregulated in LADC tumors relative to paired non-tumorous tissues and/or LADC cell lines compared with normal lung (Supplementary Figures S2 and S3). The TCGA data set validated significant but small reductions in the expression levels of these genes in tumors relative to non-tumorous tissues (Supplementary Table S2).

**Table 2:** Top 10 CpG islands significantly hypermethylated in tumors compared to paired non-tumorous tissues of 12 stage-I LADC cases

		Methylation status of CpG island		CpG island-related RefSeq gene^a^		Expression status of CpG island-related gene^b^			
Rank	CpG island	Adjusted *P*-value^c^	β-difference^d^	Gene name	Location of CpG islande	Expression in normal lung^f^	Low expression in tumors of early LADC cases^g^	Low expression in LADC cell lines^h^	Restration of expression in 5-aza-dC-treated LADC cell lines^i^
1	chr7:153583317–153585666	2.92E−05	0.2776	*DPP6*	Exon 1	**Yes**	**8/11**	**14/14**	1/3
2	chr11:125774292–125774584	3.04E−05	0.2719	*DDX25*	Exon 1	Very low	−	−	−
3	chr19:52390841–52391368	3.04E−05	0.2915	*ZNF577*	Exon 1	**Yes**	6/11	**10/14**	0/3
4	chr3:62355315–62355534	3.24E−05	0.2589	*FEZF2*	Gene body	Very low	−	−	−
5	chr1:156863415–156863711	3.26E−05	0.3692	*PEAR1*	Exon 1	**Yes**	5/11	**14/14**	0/3
6	chr15:37390175–37390380	3.81E−05	0.3249	*MEIS2*	Gene body	**Yes**	**4/11**	3/14	1/3
7	chr1:248020330–248021252	5.10E−05	0.2703	*TRIM58*	Exon 1	**Yes**	**10/11 (36/37)**	**10/14**	3/3
8	chr3:62362610–62363082	5.44E−05	0.2521	*FEZF2*	Upstream	−	−	−	−
9	chr12:103696090–103696418	5.70E−05	0.3189	*C12orf42*	Gene body	Very low	−	−	−
10	chr7:158110569–158110881	6.58E−05	0.2702	*PTPRN2*	Gene body	**Yes**	3/11	7/14	2/3

a RefSeq gene (http://www.ncbi.nlm.nih.gov/refseq/) located on or around each CpG island was selected as CpG island-related gene.

b Expression status was evaluated in 9 genes, because two CpG islands located on one gene (*FEZF2*).

c Differences between methylation levels (β-values) of CpG islands in tumors and paired non-tumorous tissues were assessed by paired *t*-test. *P*-values were adjusted with the Benjamini-Hochberg correction (False discovery rate, FDR). CpG islands were sorted by adjusted *P*-value.

d β-differences (differential methylation levels) represent the average of [(β-value of tumorous tissue) - (β-value of paired non-tumorous tissue)] in 12 stage-I LADC cases.

e Exon 1, CpG island locates within or contains exon 1 of gene; gene body, CpG island locates within or around exons other than exon 1 or introns; upstream, CpG island locates upstream of exon 1.

f Very low, expression level was undetermined by qRT-PCR in normal lung tissue (Clontech) and non-tumorous lung tissues in 11 cases with LADC.

g Low expression, > 50% reduction of relative gene expression level in tumors compared with paired non-tumorous tissues of early LADC cases. The boldface shows frequently (≥ 6/11 cases) silenced genes.

h Low expression, > 80% reduction of relative gene expression level in cell lines compared with the normal lung tissue (Clontech). The boldface shows frequently (≥ 8/14 cases) silenced genes.

i Restoration of expression, a > 4-fold (Log_2_ > 2 fold) increase in mRNA expression in 5-aza-dC-treated cells compared to paired vehicle-treated cells (control). The boldface shows frequently (≥ 2/3 cell lines) restored genes.

Finally, we conducted pharmacological re-expression experiments in LADC cell lines treated with vehicle, 5-aza-2′-deoxycitidine (5-aza-dC) and/or trichostatin A (TSA). Only *TRIM58* restoration was effectively (log_2_(fold change) > 2) and consistently (3/3 lines) observed throughout 5-aza-dC treatment (Figure 1E and Supplementary Figure S4A). In addition, the effect of 5-aza-dC treatment on *TRIM58* expression was not observed in *TRIM58*-expressing cell lines (Supplementary Figure S4B). Accordingly, *TRIM58* was identified as the most likely candidate for DNA methylation-induced silencing from the early stages of LADC, irrespective of smoking status. Decreased *TRIM58* mRNA expression in tumors relative to paired non-tumorous tissues was consistently observed even after the inclusion of 27 additional cases with various stages and smoking statuses (Figure 1C).

### Expression and CGI methylation status at the *TRIM58* promoter in LADC

**Figure 2:**
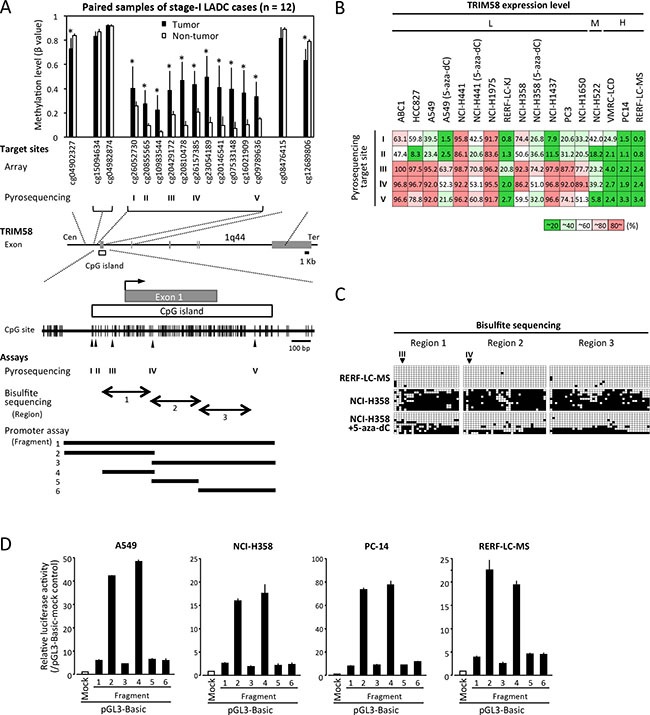
Correlation of CpG site methylation with the TRIM58 expression status in lung adenocarcinoma (LADC) cell lines (**A**) A schematic diagram of the *TRIM58* gene structure and CpG sites around exon 1 (middle), the average β-value (methylation level) of each CpG site targeted in the array-based methylation experiment involving 12 LADC cases (upper), and the CpG sites around exon 1 and the CpG island (CGI) with CpG sites targeted by pyrosequencing (arrowhead, I–V), as well as the regions analyzed via bisulfite sequencing (closed arrows, regions 1–3) and promoter assays (horizontal bar, segments 1–6). *P < 0.05 vs. paired non-tumorous tissue. (**B**) Average DNA methylation values, shown as percentages from quantitative pyrosequencing to analyze five target sites in 14 cell lines, including three cell lines treated with 5-aza-dC (triplicate experiments). Results were classified into five grades according to 20% quintiles. Cells were divided into three groups according to the expression of *TRIM58* mRNA relative to the normal lung: H, ≥ normal lung; M, < normal lung but ≥ 20% of the normal lung; L, < 20% of the normal lung. (**C**) Bisulfite sequencing of part of the *TRIM58* CGI (see Figure 2A) in RERF-LC-MS, NCI-H358, and 5-aza-dC-treated NCI-H358 cells. Open and filled squares represent unmethylated and methylated CpG sites, respectively, and each row represents the results for a single clone. Arrowheads, target sites for pyrosequencing. (**D**) Luciferase assays involving pGL3 constructs containing various fragments around the *TRIM58* CGI (see Figure 2A) in *TRIM58* non-expressing and expressing LADC cell lines (see Figure 1D). Values are expressed as the fold activation relative to pGL3-mock transfected cells (means ± standard deviations of three independent experiments).

Using methylation array data of 12 stage-I cases, we analyzed details regarding the methylation status of CpG sites around *TRIM58*. CpG sites within the CGI exhibited low levels of methylation (β value = 0.1–0.2) in non-tumorous tissues and significantly higher levels in paired tumors (Figure 2A), irrespective of the patient's smoking status (Supplementary Figure S5A). Identical results were obtained in the TCGA data set (*n* = 29, Supplementary Figure S5B).

Because almost all clinical cases exhibited decreased *TRIM58* expression, the correlation between the CGI methylation status and *TRIM58* expression status was first assessed in LADC cell lines (Figures 1D and 2B). Quantitative pyrosequencing analysis of five target CpG sites within the *TRIM58* CGI revealed low methylation levels in most CpG sites in all three LADC cell lines with higher-than-normal *TRIM58* expression levels, whereas cell lines with very low *TRIM58* expression (except RERF-LC-KJ) exhibited high levels of methylation, particularly at cg20429172 (target III) and cg26157385 (target IV). In three 5-aza-dC-treated cell lines with restored *TRIM58* mRNA expression, the methylation levels decreased to some extent at all target sites.

The methylation statuses of CpG sites within the *TRIM58* CGI around cg20429172 and cg26157385 were confirmed by bisulfite sequencing in *TRIM58*-expressing (RERF-LC-MS) and *TRIM58*-nonexpressing (NCI-H358) cells treated or not with 5-aza-dC (Figure 2C). A promoter reporter assay using various DNA fragments overlapped with the CGI in both *TRIM58*-expressing and non-expressing cell lines demonstrated that only fragments 2 and 4 containing cg20429172 and cg26157385 exhibited significant promoter activity regardless of the endogenous *TRIM58* expression status (Figure 2D), suggesting that the methylation of cis-regulatory CpG sites within fragment 4 most likely determines, at least in part, the *TRIM58* mRNA expression level.

**Figure 3:**
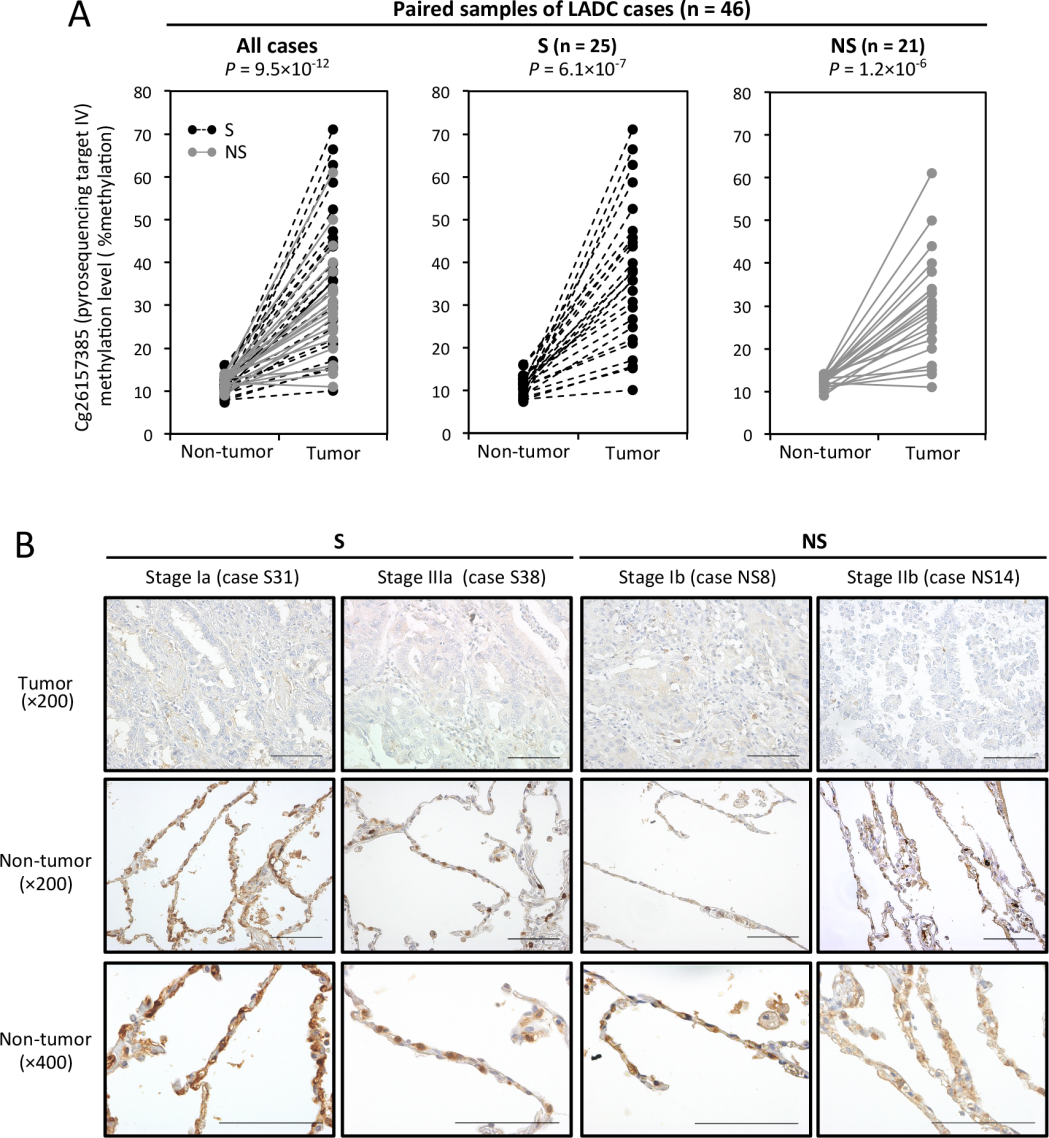
CpG site methylation and TRIM58 expression status in primary lung adenocarcinoma (LADC) tumors and paired non-tumorous tissues (**A**) Average DNA methylation values (percentages) of cg26157385 (target IV, see Figure 2A) in 46 LADC tumors and paired non-tumorous tissues from 25 smokers (S) and 21 nonsmokers (NS), as determined by quantitative pyrosequencing. Samples from the same patient are linked with dotted or straight lines. (**B**) Representative images of immunohistochemical staining for TRIM58 in LADC tumors and adjacent non-tumorous lung tissues. S and NS indicate smokers and nonsmokers, respectively. Scale bars, 200 μm.

Using normalized RNA-seq gene expression data and HumanMethylation450K BeadChip data of 17 LADC tumors and paired non-tumorous tissues from the TCGA data set, we confirmed an inverse correlation between the *TRIM58* expression and methylation levels at five pyrosequencing target sites (Supplementary Figure S5C). At those sites, particularly cg20429172 and cg26157385, mutually exclusive plotting of the expression and methylation levels was observed between tumors and non-tumorous tissues. In a larger set of our LADC cases (46 cases), pyrosequencing demonstrated the smoking status-independent, consistent tumor-specific hypermethylation of cg26157385 (Figure 3A).

To evaluate the TRIM58 protein expression status in LADC tumors and paired non-tumorous lung tissues, immunohistochemical staining (IHC) was performed (Figure 3B). In non-tumorous lung tissues, cytoplasmic TRIM58 staining was observed in alveolar epithelial cells, especially type II cells, whereas almost no TRIM58 staining was observed in LADC tumor cells, irrespective of the smoking status and tumor stage.

### TRIM58 overexpression suppresses LADC cell proliferation and tumor formation *in vitro* and *in vivo*

**Figure 4:**
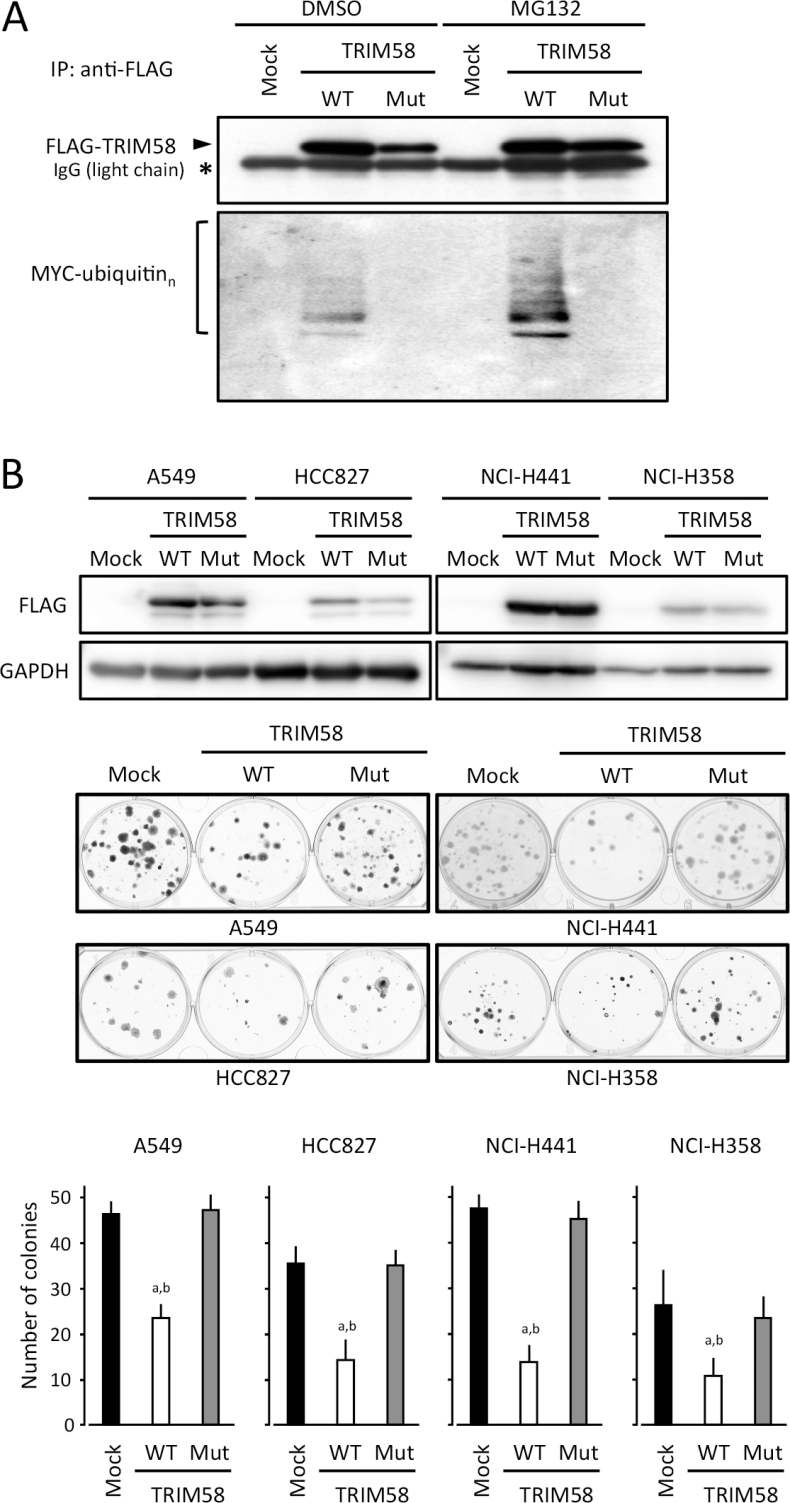
Suppressive effects of transiently transfected TRIM58 on the proliferation of lung adenocarcinoma (LADC) cells in vitro (**A**) Ubiquitin activities of wild-type and mutant TRIM58 were determined by detecting the autoubiquitination of TRIM58 proteins exogenously expressed in HEK293 cells. Cells were transiently transfected with MYC-tagged Ub and pCMV-3Tag1A (mock), pCMV-3Tag1A-wild-type TRIM58 (TRIM58-WT), or pCMV-3Tag1A-mutant TRIM58 (TRIM-Mut), cultured for 40 h, and treated with 50 μM MG132 or vehicle (DMSO) for 8 h. Cell lysates were immunoprecipitated with an anti-FLAG antibody and blotted using antibodies specific for FLAG (upper) or MYC (lower). (**B**) LADC cell lines transfected with pCMV-3Tag1A (mock), pCMV-3Tag1A-wild-type TRIM58 (TRIM58-WT), or pCMV-3Tag1A-mutant TRIM58 (TRIM58-Mut) were plated in 6-well plates and treated with 0.5 mg/mL G418 for two weeks. FLAG-tagged TRIM58 expression was confirmed by western blotting with an anti-FLAG antibody (upper). Drug-resistant colonies were stained with crystal violet and counted (middle). Columns, means ± standard deviations of four independent experiments (lower). *P < 0.05 versus mock control, Welch's *t*-test.

To investigate the biological significance of TRIM58 in the carcinogenesis of LADC, TRIM58 expression was transiently or stably restored in LADC cells lacking its endogenous expression. Because TRIM58 was silenced in most LADC cases even at early stages, we evaluated proliferation and tumor formation in those cells both *in vitro* and *in vivo*. TRIM58 is a member of the tripartite motif (TRIM)-containing protein family, which mainly comprises E3 ubiquitin (Ub) ligases containing a RING-finger domain with E3 Ub ligase activity. To determine whether the effects of TRIM58 depend on its Ub ligase activity, we simultaneously assessed a wild-type TRIM58 (TRIM58-WT) and a mutated form that lacked poly-ubiquitination activity consequent to a switch of histidine 33 to alanine within the RING-finger domain (TRIM58-Mut, Figure 4A) with the control counterparts (empty vector transfectants).

**Figure 5:**
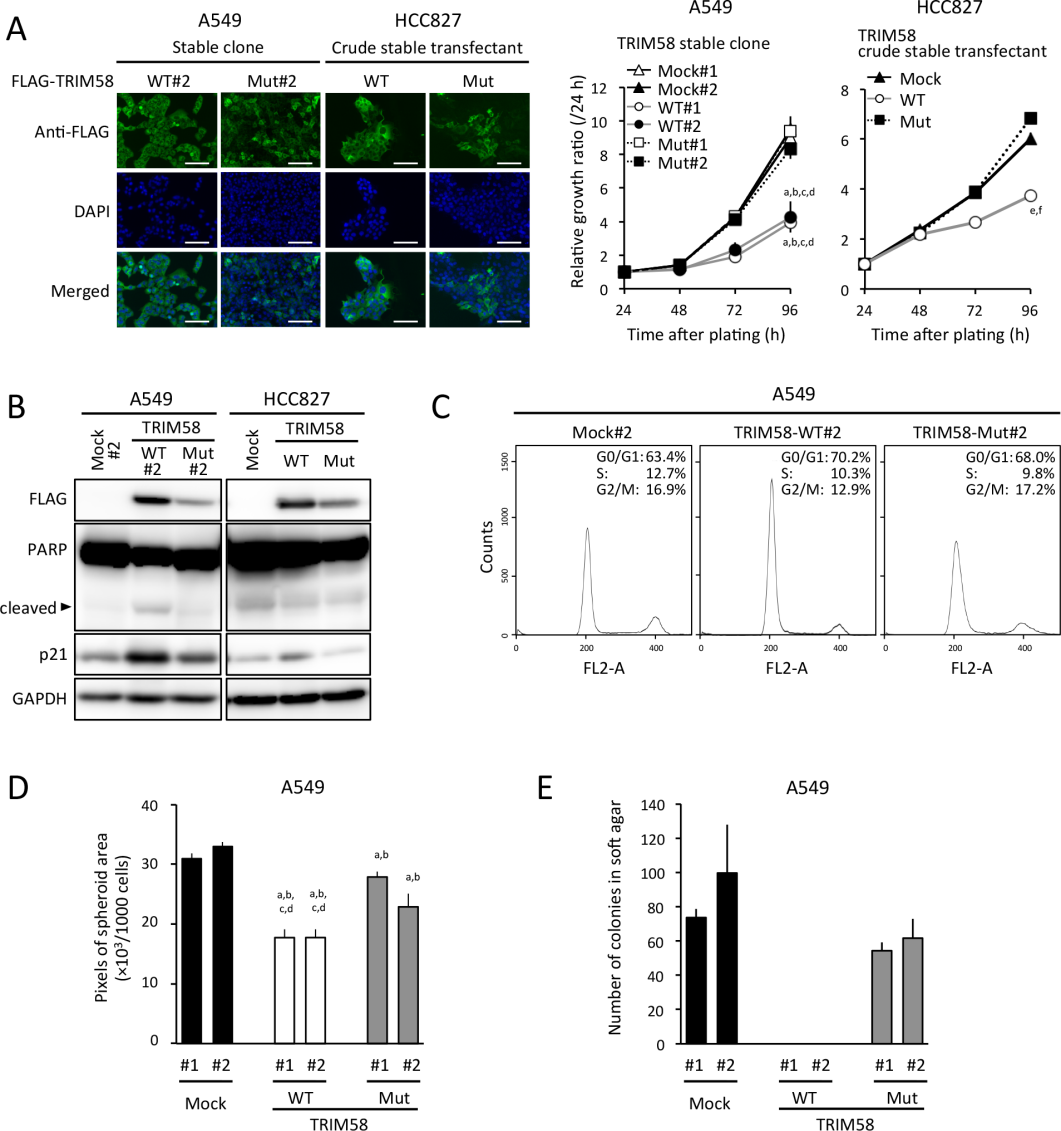
Suppressive effects of stably restored TRIM58 expression on the proliferation of lung adenocarcinoma (LADC) cells in vitro (**A**) Left, representative images of A549 or HCC827 cells engineered to stably express FLAG-tagged exogenous wild-type TRIM58 (TRIM58-WT) or mutant TRIM58 (TRIM58-Mut), detected by FIC with an anti-FLAG antibody (green). Nuclei were counterstained with DAPI (blue). Scale bars, 20 μm. Right, numbers of viable cells for each stable transfectant were assessed using a WST assay for the indicated times. Values are expressed as fold changes (mean ± standard deviation [SD], *n* = 8) relative to the respective control values (24 h). ^a,b,c,d^ *P* < 0.05 compared with the A549 mock#1, mock#2, TRIM58-Mut#1, and TRIM58-Mut#2 clones, respectively, at 96 h after plating. ^e,f^ *P* < 0.05 compared with the HCC827 crude mock and TRIM58-Mut transfectants, respectively, at 96 h after plating. (**B**) Levels of PARP, p21, and exogenous TRIM58 protein expression in each stable transfectant were analyzed by western blotting, using GAPDH as a loading control. (**C**)Representative results of the population in each phase of the cell cycle in A549 stable transfectants assessed by a FACS analysis. (**D**) Spheroid formation assay. A549 stable transfectants were seeded in ultra-low attachment 96-well round bottom plates and incubated at 37°C for seven days. Areas of the resulting spheroids were determined as described in the Materials and Methods section (mean ± SD, *n* = 8). ^a,b,c,d^ *P* < 0.05 compared to A549 mock#1, mock#2, TRIM58-Mut#1, and TRIM58-Mut#2 clones, respectively. (**E**) Colony formation assay in soft agar. A549 stable transfectants were cultured in semi-solid medium for 26 days as described in the Materials and Methods section (mean ± SD, *n* = 3).

In colony-formation assays involving transiently transfected cells, fewer colonies were produced by TRIM58-WT-transfected LADC cell lines when compared to control and TRIM58-Mut-transfected cells (Figure 4B). In an *in vitro* proliferation assay of stably transfected cells, cells expressing exogenous TRIM58-WT protein (predominant cytoplasmic location) grew significantly more slowly than mock-transfected controls and TRIM58-Mut-expressing cells (Figure 5A). Notably, p21 expression was pronounced only in TRIM58-WT-overexpressing cells, whereas a minimal effect of TRIM58-WT overexpression on PARP cleavage was observed in A549 cells and no effect on PARP cleavage was observed in HCC827 cells (Figure 5B). In a flow-assisted cell sorting (FACS) analysis in A549 cells to examine the mode of action of TRIM58 within the cell cycle, we observed an accumulation of cells in G_0_-G_1_ phase and decreases in cells in the S and G_2_-M phase without an increase in sub-G_1_ phase cells among TRIM58-WT-transfected cells relative to mock-transfected or TRIM58-Mut-transfected cells (Figure 5C). Similar results were obtained in HCC827 cells (Supplementary Figure S6), suggesting that wild-type TRIM58 contributes mainly to the arrest of LADC cells at the G_1_-S checkpoint probably through its Ub ligase activity. In addition, anchorage-independent *in vitro* 3D cell proliferation, determined via spheroid formation in low-attachment wells (Figure 5D) and colony formation in soft agar (Figure 5E), was also inhibited in TRIM58-WT-transfected A549 cells relative to mock-transfected or TRIM58-Mut-transfected counterparts.

**Figure 6:**
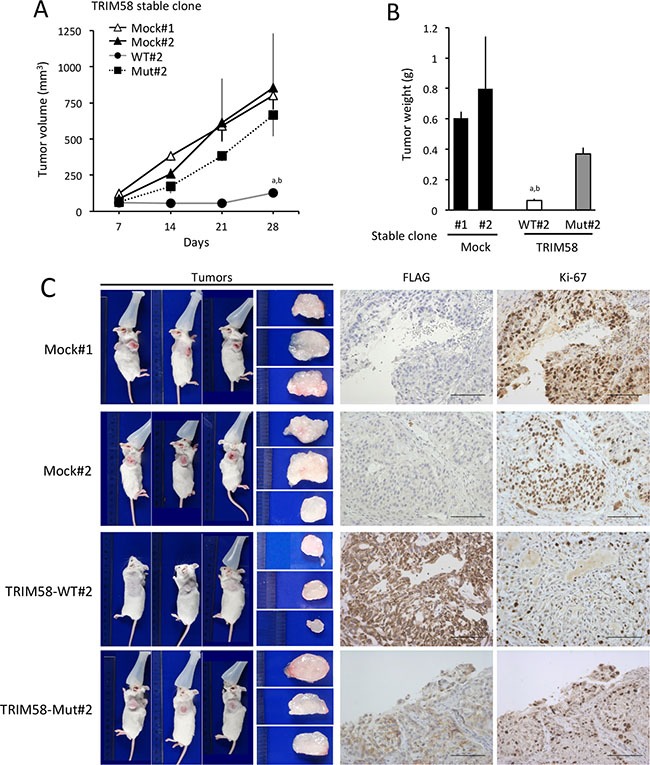
Suppressive effects of restored TRIM58 expression on the formation of A549 cell tumors in vivo (**A**) Tumor growth was assessed weekly for up to 28 days after the injection of Matrigel suspensions of each A549 stable transfectant into the flanks of severe combined immunodeficient (SCID) mice. Tumor volumes (mm^3^) were calculated as described in the Materials and Methods section. Time-dependent changes in tumor masses are shown (mean ± standard deviation [SD], *n* = 3). ^a,b^ *P* < 0.05 compared to mock#1 and mock#2, respectively. (**B**) The SCID mice used in Figure 6A were sacrificed to evaluate tumor weights on day 28 after inoculation. Mean ± SD tumor weights are shown (*n* = 3). ^a,b^ *P* < 0.05 compared to mock#1 and mock#2, respectively. (**C**) Representative tumors formed in SCID mice on day 28 after inoculation (left) and immunohistochemical analysis of FLAG-tagged TRIM58 protein (wild-type or mutant) and Ki-67 expression (right). Bars, 100 μm.

The ability of TRIM58 to suppress tumor formation *in vivo* was investigated using tumor xenograft experiments in which subcutaneous tumor growth was compared among severe combined immunodeficient (SCID) mice transplanted with different stable A549 transfectants. Restored expression of TRIM58-WT in A549 cells was associated with a reduced tumor volume and weight *in vivo* (Figure 6A, 6B, and Supplementary Figure S7). This outcome was attributed to a decrease in anchorage-dependent and/or -independent cell proliferation, as demonstrated by reduced Ki-67 positivity in TRIM58-WT-transfected cells relative to mock-transfected or TRIM58-Mut-transfected cells in resected tumors (Figure 6C).

Very recently, TRIM58 was found to facilitate erythroblast enucleation by interfering with the established ability of dynein to regulate intracellular nuclear positioning and/or microtubule structures during erythropoiesis [8]. However, the molecular mechanisms underlying the tumor-suppressive function of TRIM58 remain unknown and might be different from those involved in erythropoiesis. Indeed, our preliminary analysis demonstrated a lack of significant changes in the protein expression levels of dynein heavy chain 1 (DHC1) and intermediate chains (DIC), which were reported to be possible substrates of TRIM58 in proteasome-dependent degradation [8] between TRIM58-WT-overexpressing cells and mock-transfected or TRIM58-Mut-transfected counterparts (Supplementary Figure S8). To better elucidate the molecular mechanisms of tumor suppression by TRIM58, we performed microarray analysis to determine the effects of stable TRIM58 overexpression on the A549 cell transcriptome. We identified 1,889 probes for differentially expressed genes (DEGs) based on the criteria of a 2-fold change and an adjusted *P*-value < 0.05; specifically, 1036 and 853 probes were significantly upregulated and downregulated, respectively, in TRIM58-WT-overexpressing cells relative to mock control cells. To further identify significantly overrepresented Gene Ontology (GO) terms affected by TRIM58 expression in A549 cells, those DEGs were analyzed using The Database for Annotation, Visualization, and Integrated Discovery (DAVID) v6.7 software [9, 10]. Among the DEGs, 1562 genes (796 increased genes and 766 decreased genes) were mapped in the DAVID database. After applying a functional annotation clustering algorithm to reduce annotation redundancy, the GO analysis revealed a highly significant change in the expression of genes encoding proteins involved in the extracellular region (Cluster 1, enrichment score 7.29), extracellular matrix (ECM)/basement membrane (Cluster 2, enrichment score 7.15), and cell adhesion/cell-cell adhesion (Cluster 3, enrichment score 5.58), suggesting that TRIM58 might contribute to cell-cell and/or cell-ECM adhesion (Table 3, Supplementary Table S3).

**Table 3:** Partial list of the enriched gene ontology clusters based on the set of differentially expressed genes in TRIM58-overexpressed A549 cells

**Annotation Cluster 1**	**Enrichment Score: 7.29**			
**Category**	**Term**	**Gene count**	***P*-Value^a^**	**Adjusted P-value^b^**
GOTERM_CC_FAT	extracellular region part	133	1.0E–10	4.8E–08
GOTERM_CC_FAT	extracellular region	224	2.4E–08	3.8E–06
GOTERM_CC_FAT	extracellular space	84	5.5E–05	1.6E–03
**Annotation Cluster 2**	**Enrichment Score: 7.15**			
**Category**	**Term**	**Gene count**	***P*–Value^a^**	**Adjusted *P*–value^b^**
GOTERM_CC_FAT	extracellular region part	133	1.0E–10	4.8E–08
GOTERM_CC_FAT	extracellular matrix	64	2.4E–10	5.7E–08
SP_PIR_KEYWORDS	extracellular matrix	47	8.1E–09	1.7E–06
GOTERM_CC_FAT	proteinaceous extracellular matrix	56	3.1E–08	3.7E–06
SP_PIR_KEYWORDS	basement membrane	14	3.9E–06	6.0E–04
GOTERM_CC_FAT	extracellular matrix part	25	1.3E–05	5.9E–04
GOTERM_CC_FAT	basement membrane	19	2.9E–05	1.1E–03
**Annotation Cluster 3**	**Enrichment Score: 5.58**			
**Category**	**Term**	**Gene count**	***P*–Value^a^**	**Adjusted* P*–value^b^**
GOTERM_BP_FAT	biological adhesion	99	6.6E–08	8.1E–05
GOTERM_BP_FAT	cell adhesion	98	1.2E–07	7.3E–05
SP_PIR_KEYWORDS	cell adhesion	60	5.9E–06	6.1E–04
GOTERM_BP_FAT	cell-cell adhesion	39	9.9E–04	4.5E–02

a *P*-values were obtained by loading all differentially expressed genes (adjusted *P*-value < 0.05, upregulated and downregulated genes with > 2-fold changes) into DAVID Functional Annotation Clustering Tool (https://david.ncifcrf.gov/) to reduce annotation redundancy.

b *P*-values were adjusted with the Benjamini-Hochberg correction.

## DISCUSSION

To our knowledge, this study is the first to demonstrate the functional significance of *TRIM58* as a TSG in the context of tumorigenesis. In both our panel of LADC cases and a publicly available data set (TCGA), *TRIM58* was consistently silenced via hypermethylation in a tumor-specific manner even in the early stages of tumorigenesis and regardless of smoking status. In addition, the restoration of TRIM58 expression in LADC cells inhibited the growth of LADC cells *in vitro* and *in vivo* through its Ub ligase activity. Although the Ub ligase target substrates that underlie the tumor suppressive activity of TRIM58 remain unknown, this activity appears to associate with cell-cell and/or cell-ECM adhesion, suggesting that the inactivation of TRIM58 might contribute to the epithelial disintegration observed in early carcinogenesis. Taken together, these findings indicate that *TRIM58* likely acts as a TSG in the early stages of lung carcinogenesis, such as initiation or early tumorigenesis regardless of smoking-induced “field defects.” Indeed, the *TRIM58* CGI methylation levels in non-cancerous lung tissues of S and NS were similar (Figure 2E), suggesting that *TRIM58* may be a universal epigenetic early diagnostic marker, rather than a prognostic marker, of LADC, and may also serve as a possible preventive and/or therapeutic target through the epigenetic restoration of its expression. Further analyses of the direct Ub-ligase targets involved in the TRIM58-activated tumor suppressive pathway are needed to identify alternative preventive and/or therapeutic strategies for LADC.

The frequent inactivation of TRIM58 via hypermethylation observed even in early-stage cases from our panel of primary LADC cases was consistent with the findings of a very recent report [11] in which Diaz-Lagares *et al*. identified *TRIM58* as one of four genes that consistently exhibited CGI hypermethylation in non-small cell lung cancers (NSCLCs) relative to non-malignant lung tissues. In addition, Diaz-Lagares and colleagues found that this hypermethylation was associated with transcriptional silencing in early-stage (stage I) NSCLCs, regardless of smoking status, and identified a DNA methylation signature model based on those four genes that yielded high diagnostic accuracy for NSCLCs even in minimally invasive samples, such as bronchial fluids [11]. According to reference databases in which gene expression profiles of normal tissue have been generated using next-generation RNA sequencing technologies, such as RNA-seq Atlas (http://medicalgenomics.org/rna_seq_atlas) and RefEx (http://refex.dbcls.jp/), *TRIM58* mRNA was not detected in most normal tissues although higher expression of this gene is observed in the lung relative to the levels in various other tissues. Very recently, hypermethylation was found to correlate with decreased *TRIM58* expression in hepatocellular carcinoma (HCC) cells relative to paired adjacent liver tissues, although no functional characterization was observed in HCC cells [12]. Given the infrequency of *TRIM58* CGI hypermethylation (28.18%, 51/181 cases) and tendency of this event to correlate with unfavorable disease-free survival in HCC [12], hypermethylation appears to occur in more progressive HCC tumors. Therefore, it remains unclear whether *TRIM58* plays different tumor suppressive roles in the lung and liver.

The prevalence of CGI/promoter methylation in lung cancers, including LADC, has been reported to differ between S and NS; specifically, higher methylation of *CDKN2A*, *MGMT*, *RASSF1*, *MTHFR*, and *FHIT* promoters has been observed in S vs. NS, whereas *RASSF2*, *TNFRSF10C*, *BHLHB5*, and *BOLL* methylation is more common in NS than in S [13]. In the present study of LADC, we conducted a genome-wide screen to identify novel driver genes that contribute to early carcinogenesis following aberrant CGI methylation and consequent epigenetic activation or inactivation, regardless of the presence or absence of epigenetic field defects caused by tobacco smoking, in 12 clinically uniform stage-I LADC cases. Of the nine genes clustered around the top 10 hypermethylated CGIs in tumors, several were reported to serve as epigenetic markers of DNA hypermethylation in various malignant diseases, including LADC [9, 14–18]; however, previously described well-known methylation targets in lung cancers [13] were not included in this list. For example, *PTPRN2* hypermethylation changes had been previously reported in LADC, even in pre-neoplastic lesions or atypical adenomatous hyperplasias [14]. Frequent methylation of *PTPRN2* and *ZNF577* were reported in lung squamous cell carcinomas [15, 16]. *FEZF2* was found to be a 3p14 TSG that is frequently methylated in nasopharyngeal squamous cell carcinoma [17]. However, the effects of hypermethylation on the expression of these three genes in LADC cases were small or even opposite to the effects reported in the TCGA data set. In addition, *FEZF2* was not expressed in normal lung tissues. Furthermore, silencing of *DPP6* through hypermethylation of its promoter was reported in acute myeloid leukemia [18] and an *in vitro* neuronal differential model [19], whereas hypomethylation and subsequently increased expression of this gene have been found in colon cancer [20], suggesting that the tumor suppressive role of this gene in epithelial tumors remains unknown. Taken together, the biological significance of selected candidate genes as molecular targets for epigenetic modification therapy of LADC remains unclear, with the exception of *TRIM58*. However, the methylation statuses of these genes, which were validated in the TCGA data set except for *MEIS1* and *PTPRN2*, might be useful as early molecular diagnosis markers of LADC.

## MATERIALS AND METHODS

### Primary tissue sample collection

In total, 76 paired tumor/non-tumorous LADC sample sets were obtained from patients (39 S, 37 NS) with histologically proven primary LADC who underwent surgery at Tokushima University Hospital (Tokushima, Japan) between April 1999 and March 2015. None of the patients had received preoperative radiation, chemotherapy, or immunotherapy. A total of 12 LADC (6 S and 6 NS), 46 LADC (25 S and 21 NS), 38 LADC (18 S and 20 NS), and 63 LADC sets (28 S and 35 NS) were used for methylation screening with a HumanMethylation450K array, pyrosequencing-based methylation analysis, real-time PCR-based expression analysis, and IHC, respectively (Table 1, Supplementary Table S1).

Tumors were snap-frozen in liquid nitrogen and stored at −80°C until required for DNA and RNA analyses. Tumor specimens were characterized according to the International System of Classification of Tumors, which was based on the tumor-node-metastasis (TNM) classification of the IASLC Lung Cancer Staging Project (7th edition) [21]. Diagnoses were verified by histopathology, and only samples with at least 70% tumor cells were used in the study.

This study was performed in accordance with the principles outlined in the Declaration of Helsinki. Following the approval of all aspects of these studies by the local ethics committee (Tokushima University Hospital, approval number 2204), formal written consent was obtained from all patients.

### DNA and RNA preparation and bisulfite conversion of genomic DNA

DNA and RNA were extracted using standard methods. Bisulfite conversion of DNA was conducted using the EZ DNA Methylation Gold Kit (Zymo Research, Orange, CA, USA).

### Global methylation analysis

The HumanMethylation450K BeadChip (Illumina, Santa Clara, CA, USA) analysis was performed according to the manufacturer's instructions. The default settings of the GenomeStudio Software's DNA methylation module (Illumina) were applied to calculate the methylation levels of CpG sites as β-values (β = Intensity (methylated)/intensity (methylated + unmethylated)). The data were further normalized using a peak correction algorithm embedded in the R-package Illumina Methylation Analyzer (IMA). To identify differentially methylated CGIs between tumors and paired non-tumorous lung tissues in the discovery LADC set, median-averaged β-differences in CGI-based regions were calculated based on a β-differences matrix in which β-values of paired non-tumorous lung tissues were subtracted from those of tumors; the statistical significance of these differences was evaluated using Welch's *t*-test in IMA. Multiple testing corrections were performed using a Benjamini–Hochberg approach with significantly differential methylation, defined as a FDR-adjusted *P*-value < 0.05. The following criteria were used for differentially methylated CGIs: a β-difference > 0.25 and FDR-adjusted *P*-value < 0.05. Methylation data for the discovery cohort were deposited in the Gene Ontology Database (GEO) under accession number GSE83842.

### Cell lines

A total of 14 LADC cell lines (A549, ABC1, HCC827, NCI-H358, NCI-H441, NCI-H522, NCI-H1437, NCI-H1650, NCI-H1975, PC3, PC14, RERF-LC-KJ, RERF-LC-MS, VMRC-LCD) were obtained from the Japanese Collection of Research Bioresources (Ibaraki, Japan), RIKEN BioResource Center (Tsukuba, Japan), or American Type Culture Collection (Manassas, VA, USA) and cultured in appropriate media. To analyze the effects of DNA demethylation and/or histone acetylation on gene expression, cells were treated with 1 μM 5-aza-dC for five days and/or 300 nM trichostatin A (TSA) for the last 12 h of culture.

### Primers

Primers used in this study are listed in Supplementary Table S4.

### Quantitative real-time PCR (qRT-PCR)

qRT-PCR was performed using specific primer sets (Supplementary Table S4) and SYBR Green Master Mix (Applied Biosystems, Waltham, MA, USA) as described elsewhere [22], or TaqMan kits (Supplementary Table S4) according to the manufacturer's instructions. *GAPDH* mRNA levels were used as internal controls for normalization.

### Processing of the TCGA data set

Available RNA-seq data (IlluminaHiSeq_RNASeqV2 Level 3) and Infinium HumanMethylation450K data (Level 1) for clinical LADC specimens with clinical annotations were downloaded from TCGA Research Network (http://cancergenome.nih.gov). Gene expression data and DNA methylation data were available for 58 and 29 paired tumor/non-tumorous LADC sample sets, respectively; both types of data without outliers were available for 17 sets. The DESeq2 package was applied to raw RSEM data to perform data normalization and evaluate the differential expression status of each transcript between tumors and non-tumorous tissues [23].

To evaluate CGI methylation statuses, IDAT files were preprocessed using the R-package IMA, and the methylation level of each CG site was calculated as described above. We computed the differential gene expression and methylation statuses between tumors and paired non-tumorous tissues and evaluated statistical significance using a paired *t*-test with multiple testing corrections via the Benjamini–Hochberg approach (FDR-adjusted *P*-value).

### Antibodies

Antibodies used in this study are listed in Supplementary Table S5.

### Western blot analysis

Whole-cell lysate preparation and western blot analysis were performed as previously described [22].

### Bisulfite pyrosequencing and bisulfite genomic sequencing (BGS)

Bisulfite-treated genomic DNA was amplified using a set of primers designed with PyroMark Assay Design Software (version 2.0.01.15; Qiagen, Valencia, CA, USA; Supplementary Table S4). PCR product pyrosequencing and methylation quantification were performed on a PyroMark 24 Pyrosequencing System, version 2.0.6 (Qiagen) with sequencing primers according to the manufacturer's instructions. For BGS, bisulfite-treated genomic DNA were amplified, subcloned, and sequenced as described previously [5].

### Promoter reporter assay

Six DNA fragments around the *TRIM58* CGI were obtained via PCR with specific primer sets (Supplementary Table S4) and subsequently ligated into the pGL3-Basic vector (Promega, Madison, WI, USA). A promoter reporter assay was performed using reporter constructs as described previously [5].

### Immunohistochemical staining

Paraffin-embedded sections (thickness, 4 μm) were subjected to IHC staining using specific antibodies for each protein (Supplementary Table S5) and the Envision system (ChemMate Envision kit; Dako, Glostrup, Denmark) according to the manufacturer's instructions.. Antigen retrieval was performed by heating dewaxed and dehydrated sections in Dako Real Target Retrieval Solution, pH 9 (DAKO, Glostrup, Denmark) at 98°C for 30 min.

### Fluorescent immunocytochemical staining

Cells cultured on chamber slides were fixed, permeabilized, and stained using specific primary antibodies and fluorescently labeled secondary antibodies (Supplementary Table S5) as described elsewhere [22]. Stained cells were observed under a fluorescence microscope (LSM510; Carl Zeiss, Oberkochen, Germany).

### Expression plasmid construction

The full coding sequence of human *TRIM58* (NM_015431) was amplified via PCR (Supplementary Table S4) using cDNA prepared from normal lung RNA (Takara, Shiga, Japan). This coding sequence was then cloned into the mammalian expression vector pCMV-3Tag1A (Stratagene, La Jolla, CA, USA) with the intent to append the FLAG epitope to the NH_2_ terminus of TRIM58. The product was amplified by PCR (Supplementary Table S4) and cloned into the retroviral vector pMXs-Neo (Cell Biolabs, San Diego, CA, USA). pTRIM58-WT-FLAG harboring the H33A mutation (pTRIM58-Mut-FLAG) was generated via site-directed mutagenesis (Supplementary Table S4) with a KOD -plus- Mutagenesis Kit (TOYOBO, Osaka, Japan).

### Transient transfection experiments

A control plasmid (pCMV-3Tag1A), pTRIM58-WT-FLAG, and pTRIM58-Mut-FLAG were transfected separately into LADC cell lines or HEK293 cells using Lipofectamine 2000 reagent (Invitrogen, Carlsbad, CA, USA).

### Ubiquitin assay

HEK293T cells at < 80% confluence were co-transfected with a MYC-tagged ubiquitin-expressing plasmid and either a control plasmid (pCMV-3Tag1A), pTRIM58-WT-FLAG, or pTRIM58-Mut-FLAG for 48 h. Cells were detached by scraping in 500 μL of RIPA lysis buffer and lysed on ice for 10 min. The lysates were then centrifuged at 14,000 rpm and 4°C for 10 min, after which the supernatants (40 μg protein) were incubated for 1 h at room temperature with 20 μL of a 50% (v/v) suspension of Anti-FLAG M2 Agarose Affinity Gel (Sigma, St. Louis, MO, USA). Proteins bound to the gel were pulled down and assayed by western blotting with an anti-MYC antibody, as described above.

### Stable transfection experiments

To establish LADC cell lines that would stably overexpress wild-type or mutant TRIM58 protein, cells were infected with each TRIM58-expressing retrovirus and selected by treatment with 0.5 mg/mL G418 for four weeks. Control cells were obtained using retroviruses generated from an empty pMXs-Neo vector (mock) and packaged in PLAT-A cells. Clones were subsequently isolated, subcultured, and tested for TRIM58 overexpression by western blotting and FIC.

### Cell proliferation and cell cycle analyses

For colony formation assays, cells were transiently transfected with either mock-, pTRIM58-WT-FLAG-, or pTRIM58-Mut-FLAG-expressing plasmids (1,000 cells/well), plated in 6-well plates, and treated with 0.5 mg/mL G418 for two weeks. The expression of each TRIM58 protein variant was confirmed 48 h after transfection by western blotting. The colonies in each well were stained with crystal violet to allow quantification.

Cell growth was assessed at the indicated time after cell seeding (1 × 10^4^ cells/96-well plate) using a water-soluble tetrazolium (WST) salt assay (Cell Counting Kit-8; Dojindo, Mashikimachi, Japan) according to the manufacturer's instructions. The results are expressed as the mean absolute absorbance at the indicated time divided by the mean absolute absorbance of each sample cultured for 24 h after seeding. Cell cycle was evaluated by FACS as described elsewhere [22]. Raw cell cycle analysis data were quantified using FlowJo software (Treestar, Ashland, OR, USA).

Spheroid formation assays were performed according to a previously detailed description [22]. The areas of the resulting spheroids were determined using ImageJ software (National Institutes of Health, Bethesda, MD, USA; http://imagej.nih.gov/ij/). For soft-agar colony formation assays, a 60-mm diameter culture dish was coated with 3 ml of bottom agarose mixture (RPMI-1640/10% FBS/0.5% agarose). After the bottom layer had solidified, 2 ml of top agarose mixture (RPMI-1640/10% FBS/0.33% agarose) containing 1 × 10^5^ cells was added to each well. Triplicate cultures were incubated in a 5% CO^2^/95% O^2^ atmosphere at 37°C. At 26 days after plating, the colonies were stained with 0.03% crystal violet, photographed, and counted.

### Animal studies

The experimental use of animals described herein was approved by the Animal Care Committee of Tokushima University (approval number 13105). Five-week-old male severe combined immunodeficient (SCID) mice (CLEA Japan, Tokyo, Japan) were caged in groups of six and acclimated for one week. A suspension of cells from each stably transfected clone (2 × 10^6^ cells) was prepared in Matrigel (Corning, Corning, NY, USA) and injected subcutaneously into the bilateral flanks of SCID mice. Resulting tumor sizes were measured in two dimensions with a caliper, and volumes were calculated using the following formula: (short diameter)^2^ × (long diameter)/2. Recipient mice were sacrificed for tumor weight evaluation and protein expression analyses four weeks after tumor injection, and the growing tumors were removed and fixed with formalin in phosphate-buffered saline. Fixed xenografts were sectioned and subjected to IHC with antibodies specific for FLAG or Ki-67 (Supplementary Table S5).

### Expression array analysis and GO and pathway analyses of selected genes

LADC cell lines engineered to stably overexpress TRIM58-WT and its control counterparts were used in an mRNA analysis with a SuperPrint G3 Human GE 8 × 60k Microarray (Agilent Technologies, Santa Clara, CA, USA). RNA integrity was assessed using an Agilent Bioanalyzer 2100 (Agilent Technologies). Sample preparation, slide hybridization, and analysis were performed according to the manufacturer's standard protocols. Data were analyzed using Gene Spring GX13.0 (Agilent Technologies) and deposited to the GEO under accession number GSE85301. After applying a Benjamini–Hochberg multiple testing correction, genes expressed differentially between groups were identified as those with adjusted *P*-values < 0.05. A gene set comprising upregulated and downregulated genes with > 2-fold changes in TRIM58-WT-expressing A549 cells relative to control counterparts was analyzed using a standard enrichment analysis (DAVID Bioinformatics Resources 6.7, https://david.ncifcrf.gov/). The enrichment *P*-value calculation was performed using a Benjamini–Hochberg multiple testing correction.

### Statistical analysis

Results are expressed as means ± standard deviations (SDs). Welch's *t*-test or the Mann–Whitney *U* test was used for comparisons between two groups. The paired *t*-test and Wilcoxon signed-rank test were used for comparisons between paired samples when the data were and were not normally distributed, respectively. Multiple group comparisons were conducted using a one-way analysis of variance followed by Dunnett's post-hoc test or a Kruskal–Wallis analysis of variance followed by the Steel–Dwass post-hoc test. The relation between continuous variables was investigated by means of Pearson's correlation coefficient. Differences were assessed with a two-sided test and considered significant at a *P*-value of < 0.05. Statistical analyses were performed using R 3.2.2 (R Project for Statistical Computing, Vienna, Austria).

## SUPPLEMENTARY MATERIALS FIGURES AND TABLES








