# Supplementary material for: Frequent silencing of the candidate tumor suppressor TRIM58 by promoter methylation in early-stage lung adenocarcinoma
Source: Oncotarget. 2016 Dec 1;8(2):2890–905. doi: 10.18632/oncotarget.13761 (PMC5356850; doi:10.18632/oncotarget.13761)
Supplement: Supplementary file 1 [file oncotarget-08-2890-s001.pdf]

Frequent silencing of the candidate tumor suppressor *TRIM58* by promoter methylation in early-stage lung adenocarcinoma

Supplementary Materials

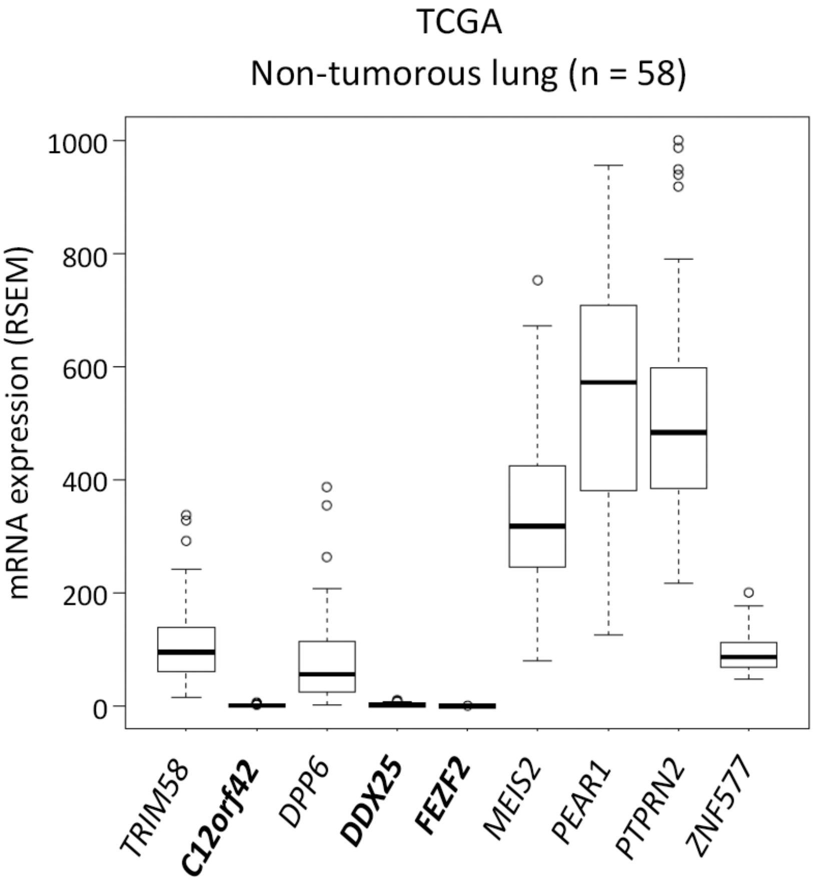

Supplementary Figure S1: Boxplot of the mRNA expression level of candidate genes in 58 non-tumorous lung samples from a TCGA data set. The y-axis represents expression levels determined by RNA-seq and quantified by RSEM. Boldface indicates genes with very low expression levels in non-tumorous lung samples.

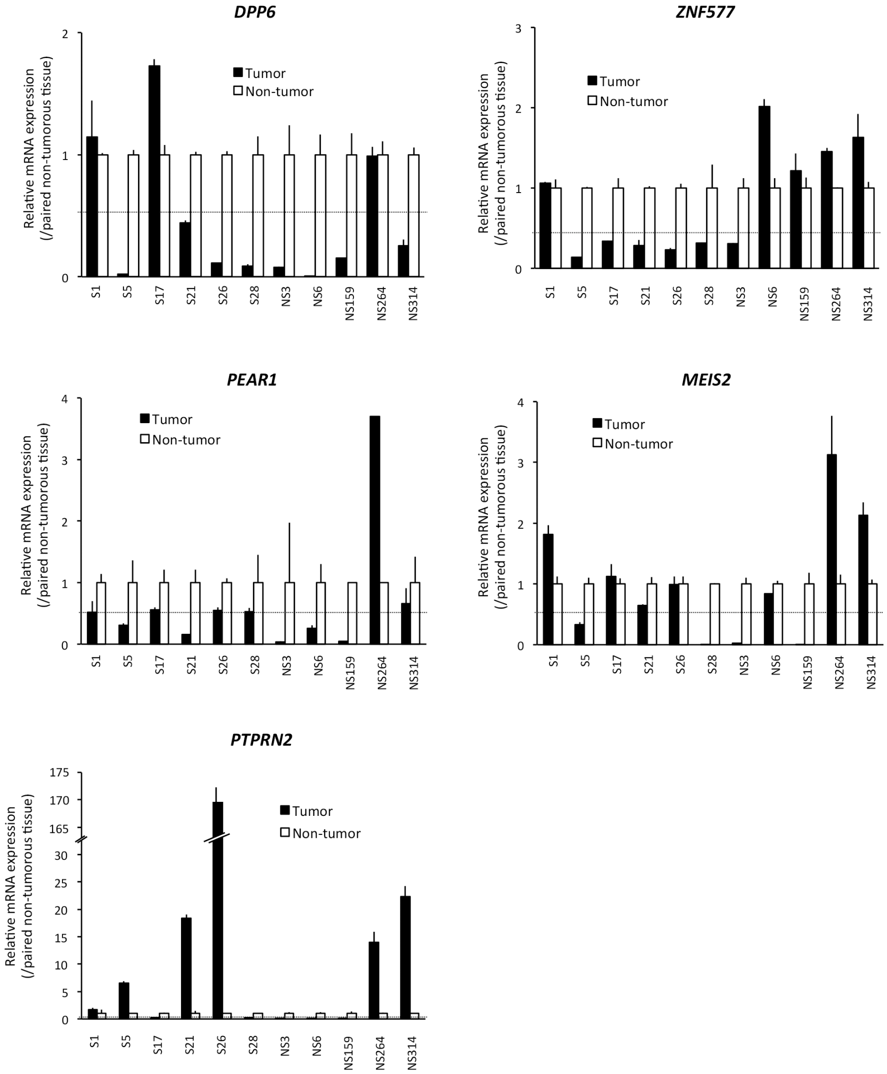

Supplementary Figure S2: Relative mRNA expression levels of candidate genes in tumor tissues vs. paired non-tumorous tissues in a panel of lung adenocarcinoma (LADC) cases used in screening experiments (*n* = 11, including six smokers and five non-smokers) evaluated by qRT-PCR and normalized to *GAPDH* (mean ± standard deviation of triplicate experiments). Dotted line indicates 0.5.

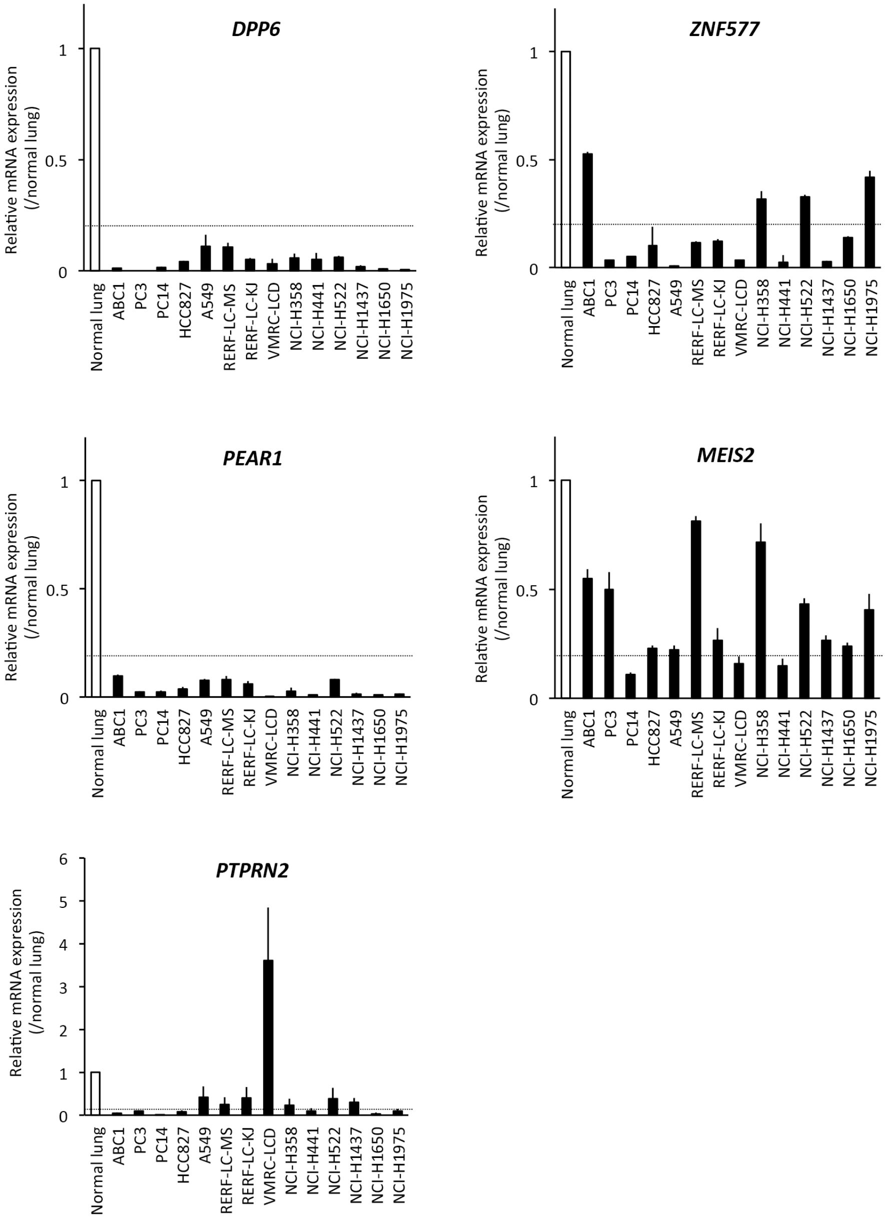

Supplementary Figure S3: Relative mRNA expression levels of candidate genes in 14 lung adenocarcinoma (LADC) cell lines vs. normal lung tissue evaluated by qRT-PCR and normalized to *GAPDH*. Dotted line indicates 0.2.

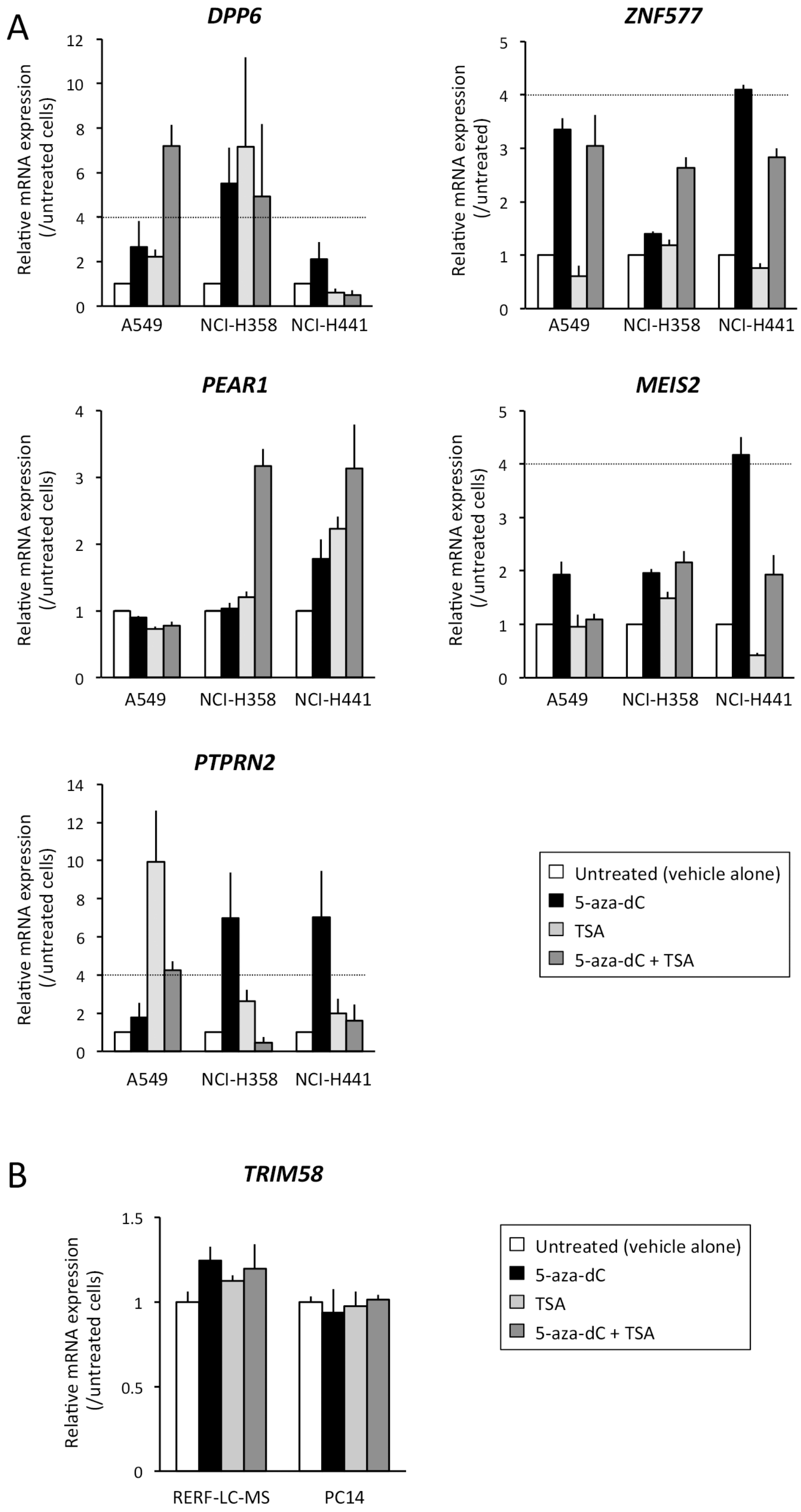

Supplementary Figure S4: (**A**) Effect of treatment with 5-aza-dC and/or trichostatin A (TSA) on the mRNA expression of candidate genes in three lung adenocarcinoma (LADC) cell lines. mRNA expression levels, which were evaluated by qRT-PCR and normalized to *GAPDH*, are shown relative to the levels in vehicle-treated control cells (mean ± standard deviation of triplicate experiments). Dotted line indicates 4 (fold change). (**B**) Effect of treatment with 5-aza-dC and/or TSA on *TRIM58* mRNA expression levels in two LADC cell lines expressing endogenous *TRIM58* with low levels of methylation in the *TRIM58* CpG sites (Figure 1D and 2B). The expression levels of *TRIM58* mRNA, which were evaluated by qRT-PCR and normalized to *GAPDH*, are shown relative to those of vehicle-treated control cells (means ± SDs of triplicate experiments).

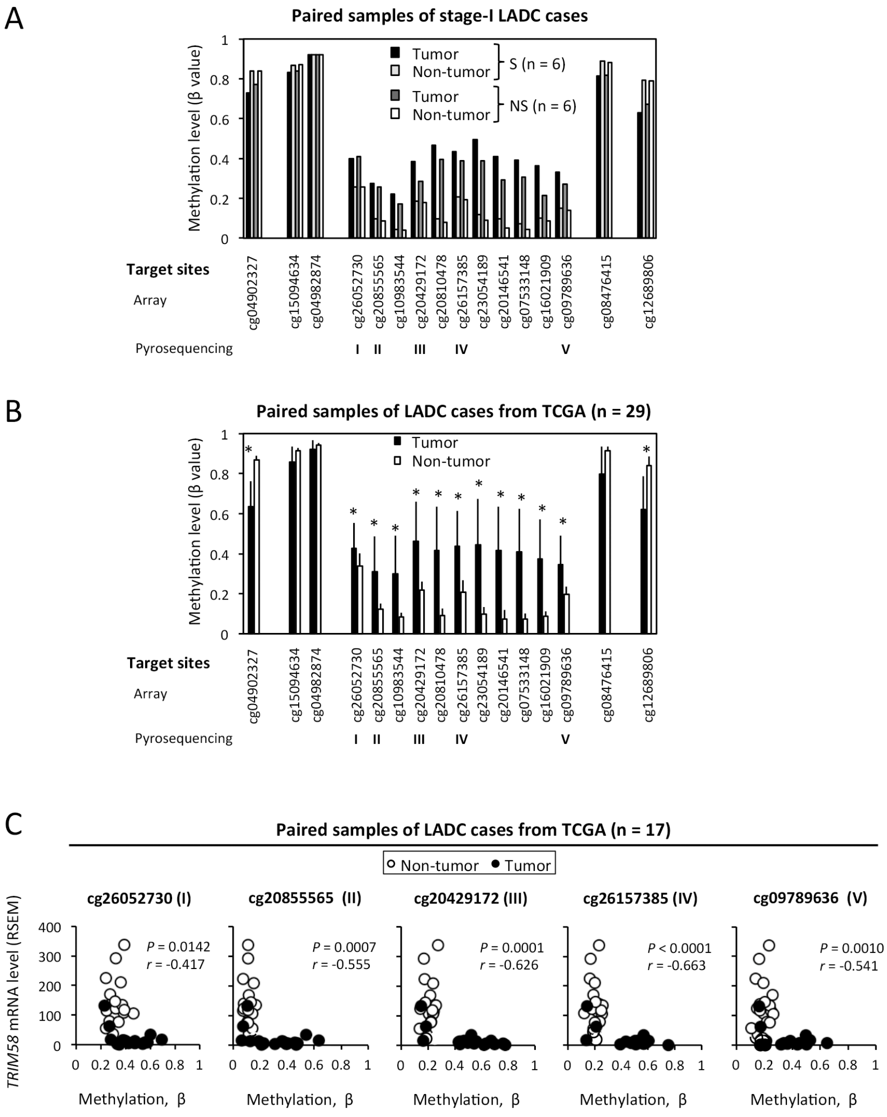

Supplementary Figure S5: (**A**) The average β-values (methylation level) of each CpG site targeted in the array-based methylation experiment (see Figure 2A) in tumors and non-tumorous tissues are shown separately for 12 lung adenocarcinoma (LADC) cases involving six smokers (S) and six non-smokers (NS). CpG sites targeted by pyrosequencing (I-V) are also shown. (**B**) The average β-values (methylation level) with standard deviations (vertical bars) of each CpG site targeted in an array-based methylation experiment involving 29 paired tumor and non-tumorous tissue samples from LADC cases obtained from a TCGA data set. *$P < 0.05$ vs. paired non-tumorous tissue. (**C**) Correlations between the methylation levels of five CpG sites within the *TRIM58* CGI and *TRIM58* mRNA expression levels in 17 paired LADC tumor and non-tumorous tissue samples obtained from the TCGA data set. The x-axis represents the β-value (methylation level) of each CpG site determined through an array-based methylation experiment (HumanMethylation450K BeadChip), whereas the y-axis represents the expression levels determined by RNA-seq and quantified by RSEM. Open and closed circles indicate non-tumorous and tumor tissues. Note that tumor and non-tumorous tissues were most clearly separated in cg20429172 and cg26157385, respectively. Roman numerals in parenthesis indicate the pyrosequencing target sites (see Figure 2A).

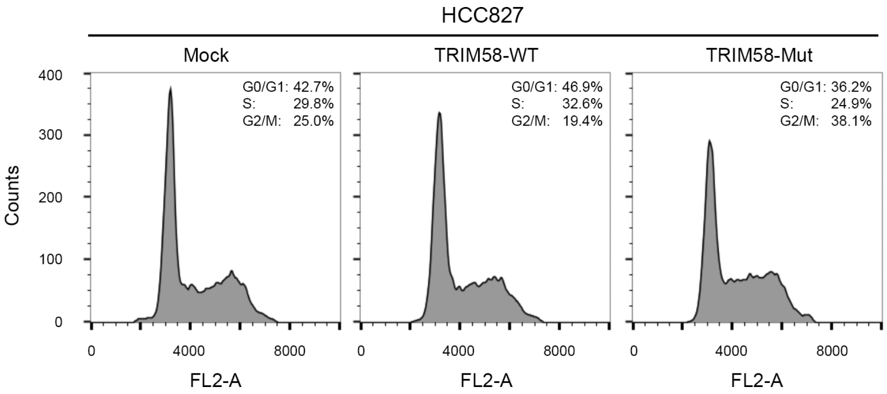

Supplementary Figure S6: Representative results of the population in each phase of the cell cycle in HCC827 stable transfectants assessed by a FACS analysis.

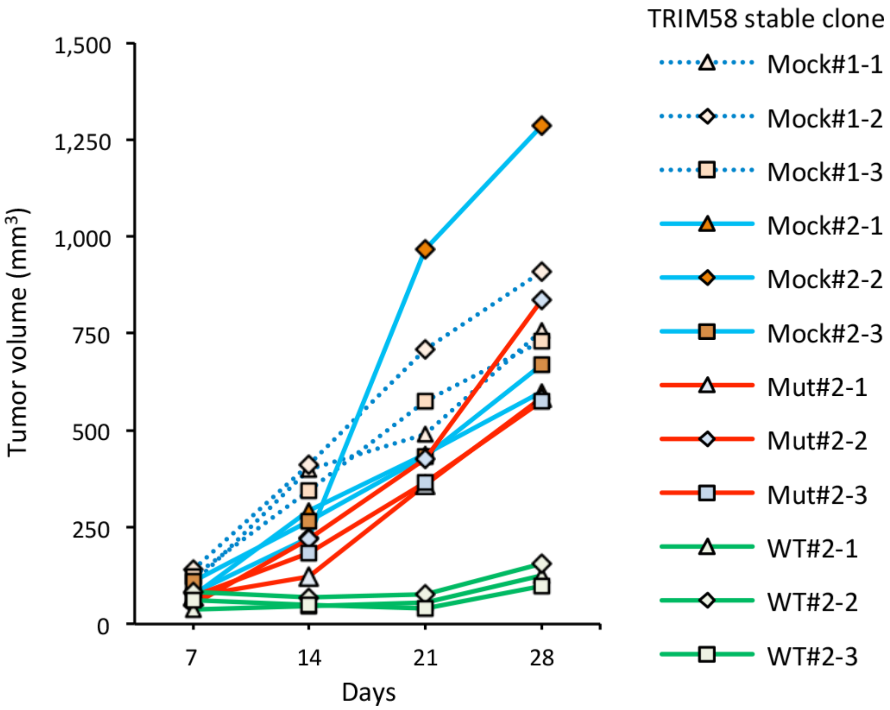

Supplementary Figure S7: ***In vivo*** **growth of each tumor after the injection of Matrigel suspensions of each A549 stable transfectant into the flanks of severe combined immunodeficient (SCID) mice.** See Figure 6A for details.

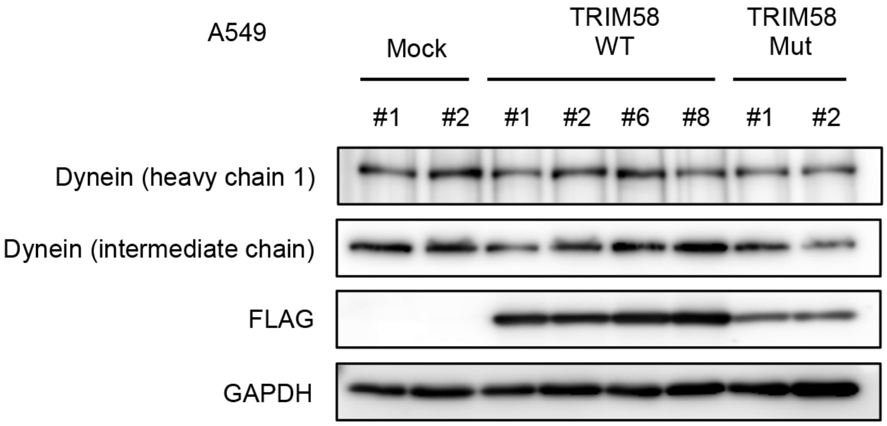

Supplementary Figure S8: Effect of stable TRIM58 overexpression on the levels of Dynein heavy chain 1 and Dynein intermediate chain proteins.

Supplementary Table S1: Clinicopathological characteristics of patients with LADC used for methylation and expression analyses of paired tumor and non-tumorous samples in this study. See Supplementary_Table_S1

Supplementary Table S2: Methylation and expression status of the 9 selected genes in tumors compared to paired non-tumorous tissues of LADC in the TCGA data set

No[a]	RefSeq gene[b]	CpG island	Methylation status in tumors compared with paired non-tumorous tissues (n = 29)		Expression status in tumors compared with paired non-tumorous tissues (n = 58)	
			Adjusted *P*-value[c]	β-difference[d]	Adjusted *P*-value[e]	Log^2(fold change)[f]
1	***DPP6***	chr7:153583317-153585666	**2.33E–07**	**0.3380**	**3.25E–57**	**−3.6742**
2	*DDX25*	chr11:125774292-125774584	**8.32E–07**	**0.2670**	7.31E–01	−0.1368
3	*ZNF577*	chr19:52390841-52391368	**4.37E–05**	**0.2190**	**3.92E–05**	−0.4389
4	*FEZF2*	chr3:62355315-62355534	**2.33E–07**	**0.2720**	2.40E–01	1.0359
5	***PEAR1***	chr1:156863415-156863711	**2.33E–07**	**0.3190**	**3.07E–68**	**−2.1425**
6	*MEIS2*	chr15:37390175-37390380	**7.56E–01**	**0.0030**	**8.52E–12**	–0.8850
7	***TRIM58***	chr1:248020330-248021252	**1.85E–05**	**0.2490**	**4.65E–41**	**–2.8577**
8	*FEZF2*	chr3:62362610-62363082	**2.33E–07**	**0.2470**	−	−
9	*C12orf42*	chr12:103696090-103696418	**2.33E–07**	**0.3820**	**3.39E–08**	1.7041
10	*PTPRN2*	chr7:158110569-158110881	1.24E–04	–0.0880	**3.52E–04**	–0.8038

[a] The order is the same as Table 2.
[b]The boldface shows genes significantly hypermethylated and silenced in tumors compare with non-tumorous tissues of LADC cases in the TCGA dataset.
[c]Differences between methylation levels (β-values) of CpG islands in tumors and paired non-tumorous tissues were assessed by paired t-test. P-values were adjusted with the Benjamini-Hochberg correction (False discovery rate, FDR). The boldface shows $P < 0.05$.
[d]β-differences represent the average of [(β-value of tumorous tissues) − (β-value of paired non-tumorous tissues)] in 29 LADC cases. The boldface shows β-differences > 0.2.
[e]Differences between mRNA expression level (RNA-Seq by Expectation-Maximization, RSEM) in tumors and paired non-tumorous tissues were assessed by paired t-test. P-values were adjusted with the Benjamini-Hochberg correction (FDR). The boldface shows $P < 0.05$.
[f]Log2(fold changes) represent the average of log2(RESMs of tumors/RSEMs of paired non-tumorous tissues) in 58 LADC cases. The boldface shows fold changes < −1.

Supplementary Table S3: List of differentially expressed genes in terms extracted as the most enriched annotation cluster in TRIM58-overexpressed A549 cells by DAVID Functional Annotation Clustering Tool. See Supplementary_Table_S3

Supplementary Table S4: List of primer sets used in PCR and qPCR. See Supplementary_Table_S4

Supplementary Table S5: List of antibodies used in this study

Antibody name	Vender[a]	ID	Purpose[b]
anti-TRIM58	Abcam	ab90362	IHC (1:300 dilution)/ FIC (1:500 dilution)/ Western blotting
anti-Ki-67	DAKO	MIB-1	IHC (1:75 dilution)/FIC (1:100 dilution)
anti-p21	Medical & Biological Laboratories	K0081-3	Western blotting
anti-p27	Medical & Biological Laboratories	K0082-3	Western blotting
anti-GAPDH	Santa Cruz Biotechnology	6C5	Western blotting
anti-PARP	Cell Signaling Technology	9542	Western blotting
anti-β-tubulin	Cell Signaling Technology	9F3	Western blotting
anti-FLAG	Sigma	M2	IHC(1:5000 dilution)/FIC(1:20000 dilution)/ Western blotting
anti-MYC tag	Cell Signaling Technology	71D10	Western blotting
anti-Dynein (heavy chain 1)	Santa Cruz Biotechnology	R-325	Western blotting
anti-Dynein (intermediate chains)	Merck Millipore	MAB1618	Western blotting
Alexa Fluor 488-labeled goat anti-mouse	Molecular Probes		FIC (1:500 dilution)
Alexa Fluor 594 goat anti-rabbit	Molecular Probes		FIC (1:500 dilution)
Alexa Fluor 594 donkey anti-goat	Molecular Probes		FIC (1:500 dilution)

[a]Santa Cruz Biotechnology, Santa Cruz, CA, USA; Medical & Biological Laboratories, Nagoya, Japan; DAKO, Glostrup, Denmark; Cell Signaling Technology, Danvers, MA, USA; Sigma, St. Louis, MO, USA; Molecular Probes, Eugene, OR, USA; Abcam, Cambridge, UK; Merck Millipore, Darmstadt, Germany.

[b]IHC, immunohistochemistry; FIC, fluorescence immunocytochemistry.
