# Supplementary material for: Frequent silencing of the candidate tumor suppressor TRIM58 by promoter methylation in early-stage lung adenocarcinoma
Source: Oncotarget. 2016 Dec 1;8(2):2890–905. doi: 10.18632/oncotarget.13761 (PMC5356850; doi:10.18632/oncotarget.13761)
Supplement: Supplementary file 2 [file oncotarget-08-2890-s002.docx]

**Table S1. Clinicopathological characteristics of patients with LADC used for methylation and expression analyses of paired tumor and non-tumorous samples in this study.**

| Patient | | | |  | TNM classification | | | |  | Sample for analysis | | | |
| --- | --- | --- | --- | --- | --- | --- | --- | --- | --- | --- | --- | --- | --- |
| ID | Age | Gender | Pack-years |  | T | N | M | Stage |  | Methylation screening (HumanMethylation450K) | Methylation (pyrosequencing) | Expression (RT-PCR) | Expression (immunostaining) |
| S1 | 68 | M | 1000 |  | 1a | 0 | 0 | Ia |  | + | + | + | + |
| S2 | 80 | M | 1200 |  | 1a | 0 | 0 | Ia |  | + | + | - | + |
| S3 | 60 | M | 1020 |  | 2a | 0 | 0 | Ib |  | + | + | + | + |
| S4 | 59 | M | 1600 |  | 1a | 0 | 0 | Ia |  | + | + | + | + |
| S5 | 74 | M | 2400 |  | 1a | 0 | 0 | Ia |  | + | + | + | + |
| S6 | 75 | M | 1000 |  | 1a | 0 | 0 | Ia |  | + | + | - | - |
| S7 | 49 | M | 1200 |  | 2a | 0 | 0 | Ib |  | - | + | - | + |
| S8 | 77 | M | 1100 |  | 2a | 0 | 0 | Ib |  | - | + | + | + |
| S9 | 77 | M | 900 |  | 1a | 0 | 0 | Ia |  | - | + | - | + |
| S10 | 52 | M | 1800 |  | 2a | 0 | 0 | Ib |  | - | + | - | + |
| S11 | 62 | M | 1230 |  | 1a | 0 | 0 | Ia |  | - | + | + | + |
| S12 | 79 | M | 2400 |  | 2a | 2 | 0 | IIIa |  | - | + | - | + |
| S13 | 70 | M | 1200 |  | 1a | 2 | 0 | IIIa |  | - | + | - | + |
| S14 | 75 | M | 2120 |  | 2a | 0 | 0 | Ib |  | - | + | - | + |
| S15 | 61 | M | 800 |  | 1b | 2 | 0 | IIIa |  | - | - | + | + |
| S16 | 65 | M | 900 |  | 2a | 0 | 0 | Ib |  | - | - | + | + |
| S17 | 64 | M | 1000 |  | 3 | 0 | 0 | IIb |  | - | + | + | + |
| S18 | 69 | M | 2000 |  | 1a | 0 | 0 | Ia |  | - | + | + | - |
| S19 | 73 | M | 2000 |  | 2a | 0 | 0 | Ib |  | - | + | - | - |
| S21 | 59 | M | 2400 |  | 3 | 0 | 0 | IIb |  | - | + | + | - |
| S22 | 75 | M | 6000 |  | 2b | 0 | 0 | IIa |  | - | + | + | - |
| S23 | 63 | M | 860 |  | 2a | 0 | 0 | Ib |  | - | + | + | - |
| S24 | 63 | M | 800 |  | 1b | 2 | 0 | IIIa |  | - | + | + | - |
| S25 | 68 | M | 1000 |  | 2a | 0 | 0 | Ib |  | - | + | + | - |
| S26 | 63 | M | 1200 |  | 1a | 0 | 0 | Ia |  | - | + | + | - |
| S27 | 61 | M | 1200 |  | 3 | 1 | 0 | IIIa |  | - | + | + | - |
| S28 | 64 | M | 1600 |  | 3 | 0 | 0 | IIb |  | - | + | + | - |
| S29 | 69 | M | 800 |  | 1a | 0 | 0 | Ia |  | - | - | - | + |
| S30 | 75 | M | 800 |  | 1a | 0 | 0 | Ia |  | - | - | - | + |
| S31 | 63 | M | 800 |  | 1b | 0 | 0 | Ia |  | - | - | - | + |
| S32 | 67 | M | 6000 |  | 1a | 0 | 0 | Ia |  | - | - | - | + |
| S33 | 62 | M | 800 |  | 2a | 0 | 0 | Ib |  | - | - | - | + |
| S34 | 72 | M | 1400 |  | 1a | 1 | 0 | IIa |  | - | - | - | + |
| S35 | 55 | M | 800 |  | 2a | 0 | 0 | Ib |  | - | - | - | + |
| S36 | 81 | M | 800 |  | 1b | 1 | 0 | IIa |  | - | - | - | + |
| S37 | 75 | M | 1100 |  | 1b | 1 | 0 | IIa |  | - | - | - | + |
| S38 | 82 | M | 2200 |  | 1a | 2 | 0 | IIIa |  | - | - | - | + |
| S39 | 76 | M | 1020 |  | 3 | 0 | 0 | IIb |  | - | - | - | + |
| S40 | 68 | M | 800 |  | 1b | 2 | 0 | IIIa |  | - | - | - | + |
| NS1 | 77 | M | 0 |  | 1a | 0 | 0 | Ia |  | + | - | - | + |
| NS3 | 57 | M | 0 |  | 1a | 0 | 0 | Ia |  | + | - | + | - |
| NS4 | 73 | M | 0 |  | 2a | 0 | 0 | Ib |  | + | - | + | + |
| NS5 | 51 | M | 0 |  | 1a | 0 | 0 | Ia |  | + | - | - | - |
| NS6 | 68 | M | 0 |  | 1a | 0 | 0 | Ia |  | + | - | + | + |
| NS7 | 74 | F | 0 |  | 1a | 0 | 0 | Ia |  | - | - | - | + |
| NS8 | 62 | F | 0 |  | 2a | 0 | 0 | Ib |  | - | - | - | + |
| NS9 | 59 | F | 0 |  | 1a | 0 | 0 | Ia |  | - | - | - | + |
| NS10 | 76 | F | 0 |  | 2a | 1 | 0 | IIa |  | - | - | - | + |
| NS11 | 57 | F | 0 |  | 2a | 2 | 0 | IIIa |  | - | - | - | + |
| NS12 | 46 | M | 0 |  | 1a | 0 | 0 | Ia |  | - | - | - | + |
| NS13 | 76 | F | 0 |  | 1a | 1 | 0 | IIa |  | - | - | - | + |
| NS14 | 74 | F | 0 |  | 3 | 0 | 0 | IIb |  | - | - | - | + |
| NS139 | 69 | F | 0 |  | 2a | 0 | 0 | Ib |  | - | + | + | + |
| NS147 | 74 | F | 0 |  | 1b | 0 | 0 | Ia |  | - | + | + | + |
| NS159 | 65 | F | 0 |  | 1a | 0 | 0 | Ia |  | - | + | + | + |
| NS180 | 63 | F | 0 |  | 2a | 0 | 0 | Ib |  | - | + | - | + |
| NS239 | 57 | M | 0 |  | 1a | 0 | 0 | Ia |  | - | - | + | + |
| NS264 | 65 | F | 0 |  | 1a | 0 | 0 | Ia |  | - | + | + | + |
| NS314 | 72 | F | 0 |  | 1b | 0 | 0 | Ia |  | - | + | + | + |
| NS335 | 78 | F | 0 |  | 1a | 0 | 0 | Ia |  | - | + | + | + |
| NS346 | 56 | F | 0 |  | 1b | 0 | 0 | Ia |  | - | + | + | + |
| NS351 | 83 | F | 0 |  | 1a | 0 | 0 | Ia |  | - | + | + | + |
| NS376 | 63 | M | 0 |  | 1a | 0 | 0 | Ia |  | - | - | + | + |
| NS382 | 62 | F | 0 |  | 1a | 0 | 0 | Ia |  | - | + | + | + |
| NS387 | 43 | F | 0 |  | 1a | 1 | 0 | IIa |  | - | + | + | + |
| NS392 | 79 | F | 0 |  | 1b | 0 | 0 | Ia |  | - | + | + | + |
| NS398 | 56 | F | 0 |  | 2a | 0 | 0 | Ib |  | - | + | - | + |
| NS463 | 59 | F | 0 |  | 1b | 0 | 0 | Ia |  | - | + | + | + |
| NS501 | 66 | F | 0 |  | 1a | 0 | 0 | Ia |  | - | + | + | + |
| NS504 | 55 | F | 0 |  | 3 | 0 | 0 | IIb |  | - | + | - | + |
| NS506 | 72 | F | 0 |  | 2a | 0 | 0 | Ib |  | - | + | + | + |
| NS556 | 70 | M | 0 |  | 2b | 2 | 0 | IIIa |  | - | - | + | + |
| NS594 | 74 | M | 0 |  | 1a | 0 | 0 | Ia |  | + | + | - | + |
| NS651 | 83 | M | 0 |  | 1a | 0 | 0 | Ia |  | - | + | - | + |
| NS676 | 71 | M | 0 |  | 2a | 0 | 0 | Ib |  | - | + | - | + |
| NS756 | 73 | M | 0 |  | 1a | 0 | 0 | Ia |  | - | + | - | + |

Samples used in HumanMethylation450K array-based methylation analysis were shaded.
