# Supplementary material for: Frequent silencing of the candidate tumor suppressor TRIM58 by promoter methylation in early-stage lung adenocarcinoma
Source: Oncotarget. 2016 Dec 1;8(2):2890–905. doi: 10.18632/oncotarget.13761 (PMC5356850; doi:10.18632/oncotarget.13761)
Supplement: Supplementary file 3 [file oncotarget-08-2890-s003.docx]

**Table S3. List of differentially expressed genes in terms extracted as the most enriched annotation cluster in TRIM58-overexpressed A549 cells by DAVID Functional Annotation Clustering Tool**

| Probe name | Gene name | Log2-fold change | Term | | |
| --- | --- | --- | --- | --- | --- |
|  |  |  | extracellular region part | extracellular region | extracellular space |
| A_33_P3341499 | WNT5A | 5.99 | + | + | + |
| A_24_P354689 | SPOCK1 | 5.19 | + | + |  |
| A_23_P83818 | COL5A1 | 4.85 | + | + |  |
| A_33_P3304668 | COL1A1 | 4.49 | + | + |  |
| A_33_P3243887 | IL11 | 4.45 | + | + | + |
| A_23_P401606 | EDIL3 | 4.28 |  | + |  |
| A_32_P32254 | COL6A1 | 3.81 | + | + |  |
| A_33_P3590259 | CXCL14 | 3.68 | + | + | + |
| A_33_P3259393 | HAPLN3 | 3.22 | + | + |  |
| A_33_P3243454 | IGFL3 | 3.20 |  | + |  |
| A_23_P55544 | CCBE1 | 3.10 |  | + |  |
| A_24_P787897 | XYLT1 | 3.09 |  | + |  |
| A_23_P148990 | HMCN1 | 3.09 | + | + |  |
| A_33_P3271657 | HHIPL1 | 2.97 |  | + |  |
| A_23_P65240 | COL4A1 | 2.95 | + | + |  |
| A_23_P213562 | F2R | 2.91 |  | + |  |
| A_23_P312150 | EDN2 | 2.90 | + | + | + |
| A_24_P406754 | LOXL4 | 2.81 | + | + | + |
| A_24_P79054 | TGFB1 | 2.78 | + | + | + |
| A_24_P158089 | SERPINE1 | 2.78 |  | + |  |
| A_24_P395814 | CGB2 | 2.76 |  | + |  |
| A_24_P395814 | CGB5, CGB8 | 2.76 |  | + |  |
| A_24_P395814 | CGB7 | 2.76 |  | + |  |
| A_23_P203115 | TMEM25 | 2.75 |  | + |  |
| A_23_P433798 | PODNL1 | 2.72 | + | + |  |
| A_23_P157865 | TNC | 2.72 | + | + |  |
| A_33_P3276207 | NXPE3 | 2.70 |  | + |  |
| A_23_P310956 | COL6A2 | 2.69 | + | + |  |
| A_33_P3319785 | COL22A1 | 2.67 | + | + |  |
| A_23_P1912 | ZP1 | 2.67 | + | + |  |
| A_33_P3308347 | ADAMTS8 | 2.61 | + | + |  |
| A_24_P402438 | TGFB2 | 2.60 | + | + | + |
| A_24_P382187 | IGFBP4 | 2.52 | + | + | + |
| A_23_P32454 | TG | 2.52 | + | + | + |
| A_23_P129695 | VASN | 2.49 |  | + |  |
| A_23_P33196 | COL5A2 | 2.49 | + | + |  |
| A_24_P7584 | LY6G5C | 2.47 |  | + |  |
| A_23_P353035 | IGFBP7 | 2.46 | + | + | + |
| A_24_P11315 | OLFML3 | 2.35 |  | + |  |
| A_33_P3400843 | C1QL4 | 2.35 |  | + |  |
| A_23_P19030 | ARSI | 2.32 |  | + |  |
| A_23_P89431 | CCL2 | 2.30 | + | + | + |
| A_23_P5983 | PLTP | 2.30 |  | + |  |
| A_23_P218505 | LHB | 2.29 |  | + |  |
| A_23_P205031 | COL4A2 | 2.28 | + | + |  |
| A_33_P3237135 | MMP2 | 2.27 | + | + | + |
| A_33_P3420816 | GDF1 | 2.26 | + | + | + |
| A_33_P3340342 | CMTM3 | 2.25 | + | + | + |
| A_23_P116235 | MDK | 2.23 |  | + |  |
| A_24_P133584 | MFGE8 | 2.21 | + | + | + |
| A_33_P3369760 | GLIPR2 | 2.18 |  | + |  |
| A_23_P121064 | PTX3 | 2.17 |  | + |  |
| A_23_P407132 | KIRREL3 | 2.12 |  | + |  |
| A_24_P88763 | LOXL3 | 2.07 | + | + | + |
| A_33_P3419696 | FGF2 | 2.03 | + | + | + |
| A_33_P3685216 | A1BG | 1.95 |  | + |  |
| A_23_P345692 | IL17D | 1.93 | + | + | + |
| A_24_P122746 | VWA1 | 1.92 | + | + |  |
| A_33_P3249872 | FBLN1 | 1.89 | + | + | + |
| A_23_P10121 | SFRP1 | 1.89 | + | + | + |
| A_23_P106602 | CRISPLD2 | 1.85 | + | + |  |
| A_32_P185637 | COL20A1 | 1.85 | + | + | + |
| A_23_P13548 | CHRDL2 | 1.82 |  | + |  |
| A_23_P951 | CFHR2 | 1.82 | + | + | + |
| A_23_P111995 | LOXL2 | 1.81 | + | + | + |
| A_23_P351667 | ADAM23 | 1.81 |  | + |  |
| A_23_P46429 | CYR61 | 1.80 |  | + |  |
| A_33_P3418125 | GLIPR1 | 1.79 |  | + |  |
| A_23_P371682 | GPC6 | 1.79 | + | + | + |
| A_33_P3216442 | COL11A2 | 1.76 | + | + |  |
| A_24_P261417 | DKK3 | 1.75 | + | + | + |
| A_23_P144071 | COL7A1 | 1.75 | + | + |  |
| A_23_P212696 | FSTL1 | 1.75 | + | + | + |
| A_23_P150693 | FJX1 | 1.73 | + | + | + |
| A_24_P220485 | OLFML2A | 1.73 | + | + |  |
| A_32_P166693 | HEG1 | 1.71 |  | + |  |
| A_23_P151778 | CMA1 | 1.70 |  | + |  |
| A_33_P3221084 | LTBP3 | 1.66 |  | + |  |
| A_23_P19663 | CTGF | 1.63 | + | + |  |
| A_23_P58588 | SLIT3 | 1.59 | + | + | + |
| A_33_P3382177 | TIMP2 | 1.58 | + | + |  |
| A_33_P3244728 | LRP2 | 1.58 | + | + | + |
| A_32_P352743 | FAM19A3 | 1.57 |  | + |  |
| A_33_P3380625 | HSPG2 | 1.54 | + | + |  |
| A_24_P142118 | THBS1 | 1.53 | + | + | + |
| A_33_P3362008 | NPPB | 1.52 | + | + | + |
| A_23_P106661 | CMTM1 | 1.48 | + | + | + |
| A_33_P3279629 | UCN2 | 1.47 |  | + |  |
| A_33_P3423570 | METRN | 1.44 | + | + | + |
| A_24_P184799 | COCH | 1.40 | + | + |  |
| A_33_P3323959 | RELN | 1.40 | + | + | + |
| A_33_P3318771 | CALCA | 1.38 | + | + | + |
| A_23_P211212 | COL18A1 | 1.33 | + | + | + |
| A_23_P386268 | C4orf26 | 1.28 |  | + |  |
| A_33_P3418209 | ITGBL1 | 1.26 |  | + |  |
| A_23_P94397 | OMD | 1.24 | + | + |  |
| A_33_P3366120 | FLNA | 1.23 |  | + |  |
| A_33_P3313810 | CELA1 | 1.22 |  | + |  |
| A_24_P365975 | COL8A2 | 1.22 | + | + |  |
| A_23_P111981 | LYNX1 | 1.21 |  | + |  |
| A_23_P76102 | GDF11 | 1.21 | + | + | + |
| A_33_P3229032 | CLEC11A | 1.20 |  | + |  |
| A_33_P3258824 | NOTCH2 | 1.19 |  | + |  |
| A_23_P147665 | OLFML1 | 1.17 |  | + |  |
| A_23_P105957 | ACTN1 | 1.15 |  | + |  |
| A_19_P00330048 | LOC102723756 | 1.14 |  | + |  |
| A_19_P00317971 | MSR1 | 1.13 | + | + | + |
| A_24_P418816 | GPX7 | 1.13 |  | + |  |
| A_23_P47181 | GPHA2 | 1.13 |  | + |  |
| A_23_P115190 | NGF | 1.10 | + | + | + |
| A_24_P48177 | ST3GAL2 | 1.08 |  | + |  |
| A_24_P358554 | BCAN | 1.08 | + | + |  |
| A_24_P135322 | NRP1 | 1.07 |  | + |  |
| A_33_P3210647 | COL16A1 | 1.06 | + | + |  |
| A_33_P3354464 | LOXL1 | 1.02 | + | + | + |
| A_33_P3294509 | CD44 | 1.00 | + | + |  |
| A_23_P26994 | GNGT2 | 1.00 |  | + |  |
| A_33_P3287631 | CTSB | -1.06 |  | + |  |
| A_33_P3308105 | GGH | -1.08 | + | + | + |
| A_23_P120883 | HMOX1 | -1.10 | + | + | + |
| A_23_P3956 | C1QTNF1 | -1.12 | + | + | + |
| A_33_P3237207 | C2orf72 | -1.13 |  | + |  |
| A_23_P214821 | EDN1 | -1.14 | + | + | + |
| A_33_P3309289 | ST3GAL4 | -1.17 |  | + |  |
| A_33_P3423365 | GSN | -1.20 | + | + | + |
| A_23_P151805 | FBLN5 | -1.20 | + | + |  |
| A_23_P31399 | PON2 | -1.23 |  | + |  |
| A_23_P8452 | LFNG | -1.26 |  | + |  |
| A_23_P62081 | SCG5 | -1.28 |  | + |  |
| A_23_P50638 | LRG1 | -1.28 |  | + |  |
| A_33_P3236392 | PVRL4 | -1.31 |  | + |  |
| A_24_P133253 | KITLG | -1.34 | + | + | + |
| A_23_P121533 | SPON2 | -1.34 | + | + |  |
| A_33_P3397763 | TNFSF9 | -1.41 | + | + | + |
| A_23_P128919 | LGALS3 | -1.42 | + | + |  |
| A_32_P186731 | ISM1 | -1.48 |  | + |  |
| A_23_P84219 | LIPH | -1.49 |  | + |  |
| A_23_P93602 | C6orf58 | -1.58 |  | + |  |
| A_23_P142389 | LSR | -1.60 | + | + | + |
| A_24_P941167 | APOL6 | -1.66 |  | + |  |
| A_33_P3391796 | NOG | -1.69 | + | + | + |
| A_23_P104798 | IL18 | -1.69 | + | + | + |
| A_23_P69537 | NMU | -1.71 |  | + |  |
| A_33_P3322363 | HMSD | -1.75 |  | + |  |
| A_23_P393620 | TFPI2 | -1.79 | + | + |  |
| A_33_P3351249 | CXCL16 | -1.80 | + | + | + |
| A_33_P3249081 | FSTL4 | -1.86 |  | + |  |
| A_33_P3295358 | ANGPTL4 | -1.88 | + | + | + |
| A_33_P3338121 | LAMB3 | -1.89 | + | + |  |
| A_23_P257003 | PCSK5 | -1.90 | + | + | + |
| A_23_P204630 | NTN4 | -1.93 | + | + |  |
| A_23_P253896 | NPNT | -1.95 | + | + |  |
| A_33_P3232688 | CSF2RA | -1.98 |  | + |  |
| A_23_P374844 | GAL | -2.00 |  | + |  |
| A_23_P157628 | DEFB4A | -2.08 |  | + |  |
| A_24_P374943 | CXADR | -2.12 |  | + |  |
| A_33_P3330264 | CXCL1 | -2.12 | + | + | + |
| A_33_P3346826 | IL32 | -2.13 | + | + | + |
| A_23_P421011 | KAZALD1 | -2.14 | + | + |  |
| A_23_P201636 | LAMC2 | -2.14 | + | + | + |
| A_33_P3262020 | C8G | -2.17 |  | + |  |
| A_33_P3384462 | THSD4 | -2.17 | + | + |  |
| A_33_P3419190 | AREG | -2.22 | + | + | + |
| A_23_P159191 | GAST | -2.33 |  | + |  |
| A_23_P94230 | LY96 | -2.37 | + | + | + |
| A_23_P61466 | CD163L1 | -2.44 |  | + |  |
| A_23_P112220 | INSL4 | -2.50 | + | + | + |
| A_23_P68487 | BMP7 | -2.50 | + | + | + |
| A_23_P43810 | LTBP1 | -2.53 | + | + |  |
| A_23_P27795 | SPINT2 | -2.58 | + | + |  |
| A_33_P3296587 | CP | -2.61 | + | + | + |
| A_23_P110531 | FST | -2.61 |  | + |  |
| A_23_P369343 | KLK8 | -2.64 | + | + | + |
| A_33_P3248903 | WNT7B | -2.64 | + | + |  |
| A_33_P3246883 | DMKN | -2.65 | + | + | + |
| A_23_P54144 | BMP4 | -2.77 | + | + | + |
| A_23_P8913 | CA2 | -2.77 | + | + | + |
| A_23_P53176 | FOLR1 | -2.80 |  | + |  |
| A_23_P75283 | RBP4 | -2.82 | + | + | + |
| A_23_P135257 | PRSS3 | -2.89 | + | + | + |
| A_33_P3284508 | CD14 | -3.04 |  | + |  |
| A_23_P501010 | COL17A1 | -3.04 | + | + |  |
| A_33_P3278649 | ENTPD2 | -3.17 | + | + |  |
| A_23_P82929 | NOV | -3.38 | + | + |  |
| A_23_P102611 | WISP2 | -3.44 |  | + |  |
| A_23_P17065 | CCL20 | -3.44 | + | + | + |
| A_33_P3227400 | COL4A4 | -3.59 | + | + |  |
| A_23_P44274 | FGA | -3.61 | + | + | + |
| A_32_P108254 | FAM20A | -3.63 |  | + |  |
| A_23_P34597 | CDA | -3.71 |  | + |  |
| A_24_P277367 | CXCL5 | -3.73 | + | + | + |
| A_23_P153571 | IGFL2 | -3.78 |  | + |  |
| A_23_P425681 | CCK | -3.90 | + | + | + |
| A_23_P118203 | ZG16B | -3.91 |  | + |  |
| A_24_P190472 | SLPI | -4.05 |  | + |  |
| A_33_P3363799 | NCAM1 | -4.10 |  | + |  |
| A_23_P21495 | FCGBP | -4.12 |  | + |  |
| A_23_P163087 | NID2 | -4.14 | + | + |  |
| A_24_P183150 | CXCL3 | -4.15 | + | + | + |
| A_23_P16523 | GDF15 | -4.16 | + | + | + |
| A_33_P3235940 | KLK6 | -4.20 |  | + |  |
| A_23_P112859 | CST1 | -4.33 |  | + |  |
| A_23_P71480 | DEFB1 | -4.35 |  | + |  |
| A_23_P67198 | CPAMD8 | -4.55 | + | + | + |
| A_23_P110253 | KIT | -4.80 | + | + | + |
| A_23_P69030 | COL8A1 | -4.92 | + | + |  |
| A_23_P210465 | PI3 | -4.94 | + | + |  |
| A_23_P30126 | FGFBP1 | -5.04 | + | + | + |
| A_33_P3708413 | MFAP5 | -5.11 | + | + |  |
| A_23_P64372 | TCN1 | -5.13 |  | + |  |
| A_23_P349416 | ERBB3 | -5.13 | + | + | + |
| A_32_P142440 | PCSK9 | -5.20 | + | + | + |
| A_24_P102650 | MUC5B | -5.36 |  | + |  |
| A_23_P136125 | FGB | -5.40 | + | + | + |
| A_23_P52761 | MMP7 | -5.42 | + | + | + |
| A_23_P19624 | BMP6 | -5.58 | + | + | + |
| A_23_P153964 | INHBB | -5.60 |  | + |  |
| A_33_P3301331 | CEACAM3 | -5.91 |  | + |  |
| A_23_P81898 | UBD | -5.96 |  | + |  |
| A_24_P322771 | TFF1 | -6.05 | + | + | + |
| A_23_P31407 | AGR2 | -6.23 |  | + |  |
| A_23_P155236 | MUC13 | -6.62 |  | + |  |
| A_23_P20484 | FGL1 | -6.63 | + | + | + |
| A_23_P69329 | HYAL1 | -7.88 | + | + | + |
