# Supplementary material for: Frequent silencing of the candidate tumor suppressor TRIM58 by promoter methylation in early-stage lung adenocarcinoma
Source: Oncotarget. 2016 Dec 1;8(2):2890–905. doi: 10.18632/oncotarget.13761 (PMC5356850; doi:10.18632/oncotarget.13761)
Supplement: Supplementary file 4 [file oncotarget-08-2890-s004.docx]

**Table S4. List of primer sets used in PCR and qPCR**

|  | Gene/primer name |  | Sequence/ID |
| --- | --- | --- | --- |
| **qPCR** | | | |
|  | *GAPDH* | Forward | 5´-AGCCACATCGCTCAGACAC-3´ |
|  |  | Reverse | 5´-GCCCAATACGACCAAATCC-3´ |
|  | *C12orf42* | Forward | 5´-CCTTGAGCAGTCCCAAGAAA-3´ |
|  |  | Reverse | 5´-GCTCGAGCTCTGTGTTACTCG-3´ |
|  | *DDP6* | Forward | 5´-ATGCAGGGGAACGTGATG-3´ |
|  |  | Reverse | 5´-GCAGTGCAATTGCTATTCCTT-3´ |
|  | *DDX25* | Forward | 5´-AGCCATGGCGTCGTTACT-3´ |
|  |  | Reverse | 5´-GGTTGGCTGAGGTTTGAAAAG-3´ |
|  | *MEIS2* | Forward | 5´-GACTGATGGCGCAAAGGTA-3´ |
|  |  | Reverse | 5´-GAACCCCTACTCCGTCCATC-3´ |
|  | *PEAR1* | Forward | 5´-CCAGACTGGAGCCTGCTT-3´ |
|  |  | Reverse | 5´-TTGGCAAGAATGGGTGCT-3´ |
|  | *PTPRN2* | Forward | 5´-GCCTTCACCTCTGGGAGATT-3´ |
|  |  | Reverse | 5´-GAGGGTATGAATCCGTGCTC-3´ |
|  |  |  |  |
| **TaqMan gene expression assay** | | | |
|  | *TRIM58* | FAM | Hs00296057_m1 |
|  | *FEZF2* | FAM | Hs01115572_g1 |
|  | *ZNF577* | FAM | Hs00261929_m1 |
|  | *GAPDH* | FAM | Hs02758991_g1 |
|  |  |  |  |
| **Bisulfite (BS) PCR** | | | |
|  | BS-*TRIM58*-Region1 | Forward | 5'-ATATAGGGAGYGTTTGAGTGG-3' |
|  |  | Reverse | 5'-TCCTACAAAAAATCCAAACACA-3' |
|  | BS-*TRIM58*-Region2 | Forward | 5'-TGTGTTTGGATTTTTTGTAGGA-3' |
|  |  | Reverse | 5'-CAAAAACRACTCAAATCCTC-3' |
|  | BS-*TRIM58*-Region3 | Forward | 5'-AGGATTTGAGTYGTTTTTG-3' |
|  |  | Reverse | 5'-CAAAAAAACCCACACTAAATAA-3' |
|  |  |  |  |
| **Pyrosequencing for *TRIM58*** | | | |
|  | cg26052730 & cg20855565 | Forward | 5'-ATTGTTAGTGAAAATAGTATATGAGGAGGG-3' |
|  |  | Reverse | Biotin-5'-AACCAATAATATCCCCTAACTTCAATTCAAA-3' |
|  |  | Sequence | 5'-GTTAGAAATGTTTAGT-3' |
|  | cg20429172 | Forward | 5'-AGAGGTTTGTATAGGGGGAGGA-3' |
|  |  | Reverse | Biotin-5'-AAAAATTCCAATTAAAAATACCCAATTTCCT-3' |
|  |  | Sequence | 5'-TAAGTTTTAGGAAAGTA-3' |
|  | cg26157385 | Forward | 5'-GTAGAATTAGGAGATGTATTTGAGGTAGAAG-3' |
|  |  | Reverse | Biotin-5'-CCATACCCCATATACCTAAATTTCCTACAAA-3' |
|  |  | Sequence | 5'-GGTAGAAGTTGTGG-3' |
|  | cg09789636 | Forward | 5'-GATAGGAGAGTGTTTGGGGGA-3' |
|  |  | Reverse | Biotin-5'-TCTCTTTACCTTAACTATAATAACCCCCT-3' |
|  |  | Sequence | 5'-GTGTTTGGGGGAG-3' |
|  |  |  |  |
| **Construction of expression plasmids for recombinant TRIM58** | | | |
|  | Full coding TRIM58 for pCMV-3Tag1A | Forward^a^ | 5'-TTGAATTCGCCTGGGCGCCGCCCG-3' |
|  |  | Reverse^b^ | 5'-TTTCTCGAGTTTTAGAGATGATCATCTCTTACA-3' |
|  | Full coding TRIM58 for pEGFP-C | Forward^b^ | 5'-TTTCTCGAGCCTGGGCGCCGCCCG-3' |
|  |  | Reverse^a^ | 5'-TTGAATTCTTTTAGAGATGATCATCTCTTACA-3' |
|  | FLAG-tagged TRIM58 for pMXs-Neo | Forward^b^ | 5'-CCTTCTCGAGCCACCATGGATTACAAGGA-3' |
|  |  | Reverse^d^ | 5'-CCTTGCGGCCGCTTAGATCATCTCTTACAT-3' |
|  | Mutagenesis (H33A) in TRIM58 | Forward^e^ | 5'-ACTGCGGCGCCAGCTTCTGCCT-3' |
|  |  | Reverse^e^ | 5'-CAGAAGCTGGCGCCGCAGTCCA-3' |
|  |  |  |  |
| **Construction of reporter promoter plasmids for *TRIM58*** | | | |
|  | *TRIM58*-promo-F1(-370) | Forward^f^ | 5´-TTACGCGTAGTGAGCCGAGATCGCGCC-3´ |
|  | *TRIM58*-promo-F2(-122) | Forward^f^ | 5´-TTACGCGTCCTGAGTGGTGGCTTTTCAC-3´ |
|  | *TRIM58*-promo-F3(+101) | Forward^f^ | 5´-TTACGCGTTGTGCCTGGATTTCCTGCAC-3´ |
|  | *TRIM58*-promo-F4(+350) | Forward^f^ | 5´-TTACGCGTAGGACCTGAGCCGCTTCTG-3´ |
|  | *TRIM58*-promo-R1(+762) | Reverse^b^ | 5'-TTTCTCGAGTTTCTCGAGTGACCGGCTCCTGCAGGAA-3´ |
|  | *TRIM58*-promo-R2(+130) | Reverse^b^ | 5´-TTTCTCGAGCGCATCGGGAGAAACCAGC-3´ |
|  | *TRIM58*-promo-R3(+359) | Reverse^b^ | 5´-TTTCTCGAGAAGCGGCTCAGGTCCTC-3´ |

^a^ *Eco*RI site is underlined

^b^ *Xho*I site is underlined

^c^ *Sal*I site is underlined

^d^ *Not*I site is underlined

^e^ Replaced codon is underlined

^f^ *Mlu*I site is underlined
